# Absorption Coefficients of Phenolic Structures in Different Solvents Routinely Used for Experiments

**DOI:** 10.3390/molecules26154656

**Published:** 2021-07-31

**Authors:** Julia A. H. Kaeswurm, Andreas Scharinger, Jan Teipel, Maria Buchweitz

**Affiliations:** 1 Department of Food Chemistry, Institute of Biochemistry and Technical Biochemistry, University of Stuttgart, 70569 Stuttgart, Germany; julia.kaeswurm@lc.uni-stuttgart.de; 2 Chemisches und Veterinäruntersuchungsamt Karlsruhe, Weißenburger Str. 3, 76187 Karlsruhe, Germany; andreas.scharinger@cvuaka.bwl.de (A.S.); jan.teipel@cvuaka.bwl.de (J.T.)

**Keywords:** polyphenols, anthocyanins, absorption coefficient, q-NMR

## Abstract

Phenolic structures are of great interest due to their antioxidant properties and various postulated benefits on human health. However, the quantification of these structures in fruits and vegetables, as well as *in vivo* or *in vitro* experiments, is demanding, as relevant concentrations are often low, causing problems in exactly weighing the respective amounts. Nevertheless, the determination of used concentrations is often a prerequisite for accurate results. A possibility to quantify polyphenol is the use of UV/vis spectroscopy. Therefore, the absorption coefficients of selected phenolic structures were determined in three different solvents relevant for polyphenol research (water/methanol (50/50, *v/v*), water, and phosphate buffer at *pH* 7.5). To confirm the values based on weight and to avoid errors due to impurities, hygroscopic effects, and inadequate balance care, the mass concentrations were additionally determined by quantitative NMR (q-NMR). The coefficients presented in this article can help to quickly and easily determine accurate concentrations in a laboratory routine without wasting the often-precious standard compounds.

## 1. Introduction

For polyphenols and water-soluble secondary plant substances, many positive health effects have been proposed [1]. Besides identifying and quantifying phenolic structures in food [2,3,4,5], current research attempts to prove the postulated effects on human health have been performed by the use of in vivo and in vitro experiments [6,7,8,9]. Some previous investigations have focused on the holistic evaluation of the effects of polyphenolic extracts but not on the individual substances and their properties [10,11]. Our aim was to provide reliable data as a basis for further in-depth research into the quantification of individual phenolic structures and clarification of their interaction mechanisms. Quantification in biological samples and experiments into the effects and the biochemical mechanisms require stock solutions and dilutions with defined and precisely determined concentrations.

Particularly for physiologically relevant concentrations, the exact weighing is problematic. Isolated compounds might contain impurities of substances, which are not detectable by routinely applied methods such as HPLC-DAD-MS. In addition, commercially available phenolic standard compounds, with the exception of simple hydroxyl cinnamic acids, are cost-intensive and exhibit a limited shelf life in solution. Moreover, more hydrophobic phenolic structures can be dissolved in aqueous media (electrolyte solutions or buffers) only to a limited extent. Micro-balances fit to weigh sub-milligram amounts of substances are cost-intensive and require a strictly controlled environment. In addition, systematic errors can occur if they are not adequately maintained, serviced, and calibrated. Apart from general individual weighing errors, the lyophilized phenolic powders are often hygroscopic, which leads to corresponding weighing inaccuracies.

**Figure 1:**
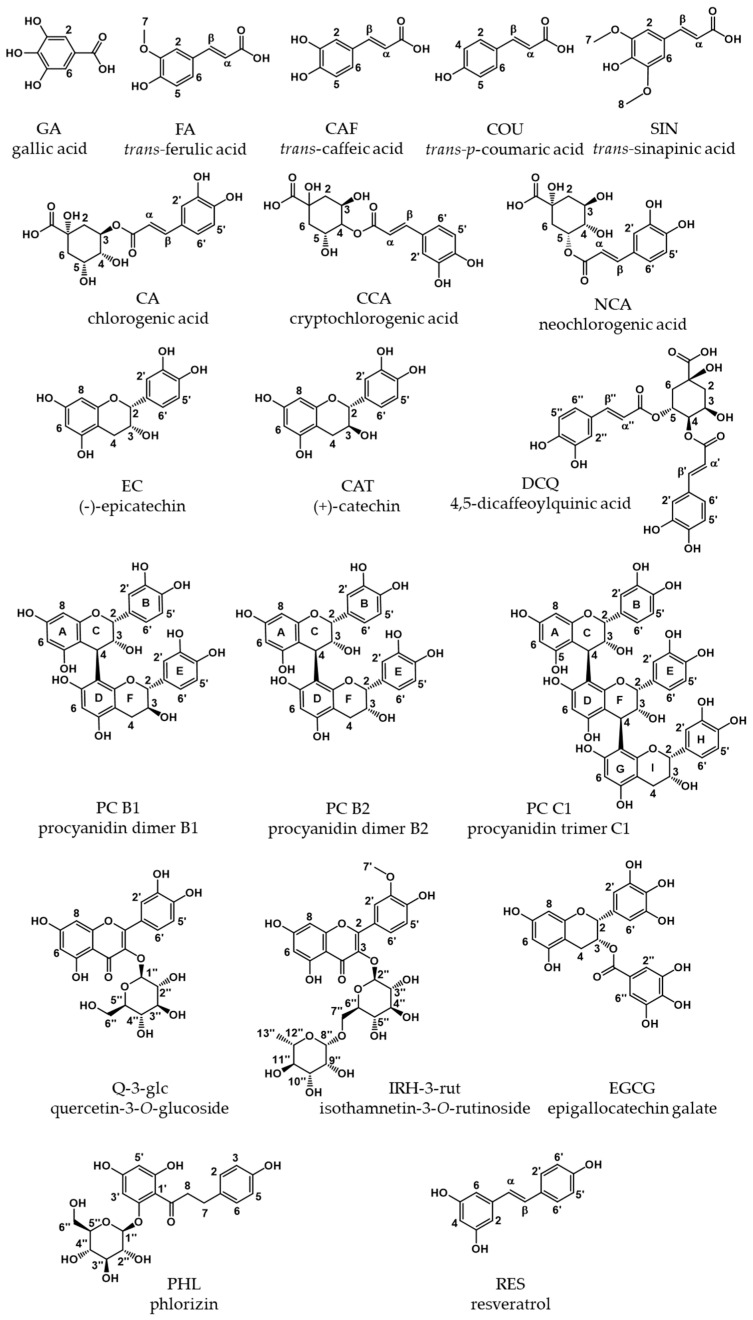
Overview of phenolic compounds investigated.

As polyphenols are aromatic substances, it is possible to determine their absorption at 280 nm by means of UV spectroscopy. According to the Bouguer–Lambert–Beer law, a substance’s light absorption is proportional to its concentration in a given solvent; however, this is limited to a substance- and solvent-specific maximum concentration. Particularly, phenolic compounds tend to form supramolecular structures at higher concentrations in aqueous solutions [12], which limit the linear proportionality [13]. With the expansion of the conjugated π–electron system, the maximum absorption shifts from 280 nm to higher wavelengths (bathochromic effect). Furthermore, the wavelength might shift when different solvents are used, due to *pH*-dependent equilibria. Therefore, we determined absorption coefficients for some phenolic structures (Figure 1) in three different solvents: water, aqueous methanol (50/50 *v/v*), and aqueous phosphate buffer at *pH* 7.5 at *λ*_max_, the individual wavelength of maximum absorption, and at 280 nm for comparison.

As the determination of absorption coefficients requires a reliable and confirmed concentration determination, we compared the data based on weight with concentrations determined by quantitative NMR (q-NMR). In recent years, q-NMR has been proven as a fast, reliable, sample saving and nondestructible absolute method to determine concentrations [14,15,16,17]. The quantifications performed by q-NMR are based on specific proton signals of the different substances.

## 2. Results 

The following tables combine the results we found. Table 1 lists the extinction coefficients determined at the substances’ individual wavelengths of maximum absorption (*λ*_max_). In Table 2 the extinction coefficients measured at the common wavelength *λ* = 280 nm are given. Table 3 shows the extinction coefficients determined in strongly acidic aqueous solution, both at *λ*_max_ and at *λ* = 280 nm. 

## 3. Discussion

**Figure 2:**
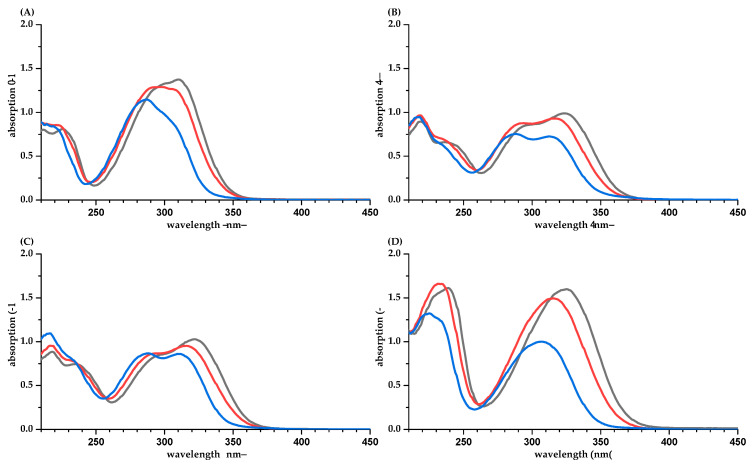
UV spectra of hydroxycinnamic acids in water/methanol (50/50, *v/v*, black), water (red), and phosphate buffer *pH* 7.5 (blue). (**A**), coumaric acid; (**B**), caffeic acid; (**C**), ferulic acid; (**D**), sinapinic acid. Concentrations are different for the four hydroxycinnamic acids but similar among the solvents.

The absorption coefficients in methanol/water for COU, CAF, FER, and SIN are comparable with the values found by Rubach with 18,800, 15,800, 13,300, and 16,700 L·mol^−1^·cm^−1^, at *λ*_max_, respectively [18]. The structures of hydroxycinnamic acids are *pH*-dependent. In water, the *pH* values are concentration-dependent and range from 4.9 to 5.2 (Table A1). In buffer, the carboxylic group tends to dissociate, which explains the hypsochromic shifts in *λ*_max_ and the decrease in absorption in phosphate buffer due to an increased formation of the negatively charged structures (Figure 2). The *pK*_a_ values, calculated by ChemAxon and listed in the HMDB data bank [19], are in a similar range with 4.00, 3.64, 3.77, and 3.61 for COU, CAF, FER, and SIN, respectively, and explain the increased bathochromic shifts. The values for the absorption coefficient calculated with a concentration based on balance or q-NMR are in a good agreement.

The absorption coefficients for chlorogenic acid derivatives are independent of the ester position and the solvent (Table 1 and Table 2, Figure 4A). Surprisingly, esterified with quinic acid, the absorption coefficient is roughly 25% higher compared to free CAF. The significantly lower absorption at 280 nm underlines the importance to quantify these phenolic compounds separately at their individual absorption maxima or summarized at 320 nm. Our values determined in water and methanol/water are in good agreement with a former study by Rubach. Here, 19,500, 18,000, and 18,400 L·mol^−1^ cm^−1^ were found for chlorogenic (3′), neochlorogenic (4′), and cryptochlorogenic (5′) acid [18]. The UV spectra of chlorogenic acids are not significantly influenced by the solution’s *pH* values (Figure 4A). In water, the *pH* values of the isomers are significantly different, with 5.0 (CA), 4.6 (CCA), and 5.6 (NCA) (Table A1). However, the carboxylic group of the quinic acid with a *pK*_a_ of 3.3 [19] is widely distanced from the aromatic system, which is responsible for the absorption in the UV range. DCQ contains two independent CAF units and, therefore, the absorption should be doubled. However, the data are closer to the sum of the absorption of a chlorogenic acid and CAF.

Our values for CAT and EC are in agreement with the literature. A value of *ε* = 3988 L·mol^−1^·cm^−1^ has been reported for CAT and EC in methanol at 280 nm [20]. The absorption coefficients for the two dimers (PC B1 and B2) are in a similar range and are roughly doubled compared to the monomers. The trimer PC C1 follows the same trend comparing the data obtained by balance. In pure water and, in particular, in phosphate buffer, the absorption is reduced. In water, the *pH* value of all flavanols investigated is about *pH* 6 (Appendix A Table A1) and we interpret this more as an effect of the solvent’s dielectric constant, than an effect of the *pK*_a_ (*pK*_a_ CAT/EC = 9) [19]. The q-NMR data of the procyanidins are suspicious. Due to the formation of rotamers, quantification of the procyanidins by NMR is hampered. Fortunately, in methanol/water, the sum of the signals for the six protons of the B- and E-ring and the two diastereomeric protons at position F 4 are suitable to quantify the dimers, ignoring the different ratios of the two rotamers [21,22] (Supplementary Material). For the trimer PC C1, the number of rotamers is even higher (up to 4) [22,23], significantly influencing signal intensity and, therefore, integration.

**Figure 3:**
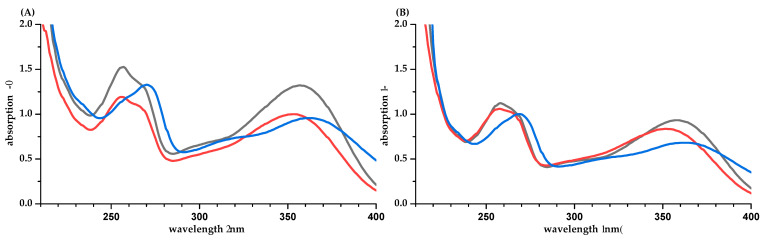
UV-spectra of IRH-3-rut (**A**) and Q-3-glc (**B**) in water/methanol (50/50, *v/v*, black), water (red), and phosphate buffer *pH* 7 (blue). Concentrations are different for the two flavonoids but similar between the solvents.

The UV spectra of the flavonoids IRH-3-rut and Q-3-glc show two maxima around 260 nm (B-ring) and around 360 nm (A and C-ring) (Figure 3). Gitelson et al. reported an absorption coefficient for quercetin-rutinoside of 25,400 L·mol^−1^·cm^−1^ at 358 nm in 80% aqueous methanol [24]. This is higher than the value of 21,515 ± 964 L·mol^−1^·cm^−1^ found in this study for Q-3-glc (based on q-NMR, Table 1). The absorption coefficient calculated with the mass concentration *γ* based on weight is markedly reduced. Due to the unknown purity of Q-3-glc and problems with precipitations, we rather trust the value based on NMR. The *pH* of the aqueous solution is 6.6 and 6.0 for IRH-3-rut and Q-3-glc, respectively. Both compounds have *pK*_a_ values of 6.4 [19], and an increased formation of the deprotonated structure is obvious, comparing the spectra in water and buffer at *pH* 7.5. The most acidic position is the hydroxyl group at position 7 (A-ring). However, due to mesomeric effects, the negative charge is transferred to position 4′ in the B-ring and a bathochromic shift of *λ*_max_ is observed for both maxima.

**Figure 4:**
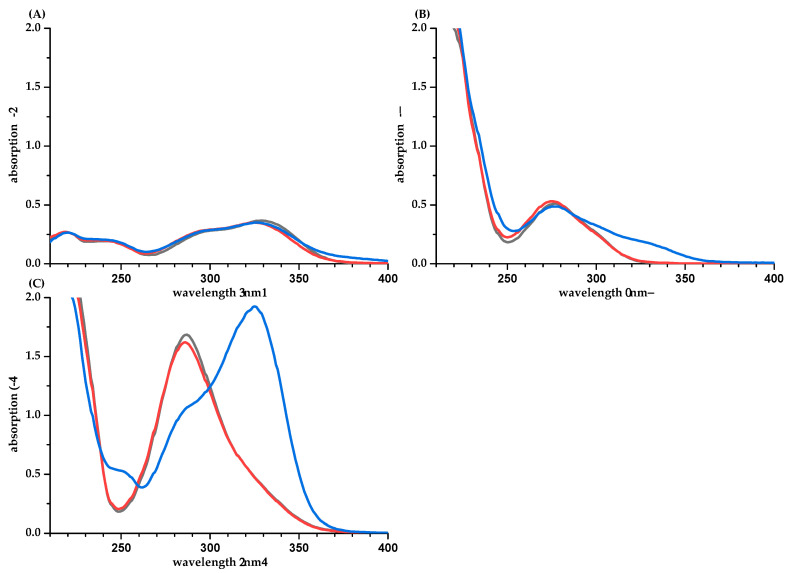
UV spectra of CA (**A**), EGCG (**B**), and PHL (**C**) in water/methanol (50/50, *v/v*, blue), water (orange), and phosphate buffer *pH* 7.5 (gray).

For EGCG (*pK*_a_ 7.99) [19], the UV absorption spectra in water (*pH* value is 6.0) and phosphate buffer are different (Figure 4B). However, the impact on the absorption coefficient is marginal. For PHL, a strong bathochromic shift and an increased absorption are observed in phosphate buffer (Figure 4C). This is due to the increased formation of the deprotonated, anionic PHL species (*pK*_a_ 7.87 [19], *pH* in water is 6.0).

For anthocyanins, a wide variety of absorption coefficients are available in the literature, and some of them have been summarized by Giusti and Wrolstad [25]. However, the data vary in the wavelength of absorption and the solvent used. In particular, the *pH* value plays an important role for anthocyanins due to the *pH*-dependent equilibration between the red flavylium cation and the colorless hemicetal. Therefore, *pH* values were checked for all anthocyanidin NMR dilutions to be *pH* ≤ 1.1. Nevertheless, our values for the absorption coefficients differ significantly between the calculations based on the balance and q-NMR (16–40% higher in the calculation based on q-NMR, Table 3, Supplementary Material Table S1). Despite difficulties in weighing the hygroscopic anthocyanidins, we assumed a systematic underestimation by q-NMR. Data from the literature, in particular, the value of 26,900 L·mol^−1^·cm^−1^ for CYD-3-glc [25], support this. Therefore, we diluted two acidic (0.1% DCl) aqueous stock solutions of DPD-3-glc (1 g/mL, 1.6 g/mL) with potassium chloride buffer *pH* 1 and methanol-d_4_ and determined the solutions’ mass concentrations by q-NMR. A significant concentration difference (~20%) was observed between samples in buffer at *pH* 1 and acidic methanol-d_4_/D_2_O (50/50, *v/v*) (Supplementary Material Table S2, Figure S1). Excluding the protons at position 6 and 8 (A-Ring), the mass concentration determined in methanol-d_4_/D_2_O was similar to the mass concentration calculated by weight. Reduced integrals for protons at these positions have also been reported for other flavonoids [21].

The partial NMR excitation due to insufficient relaxation delay was checked by comparing spectra recorded with shorter vs. longer recycle delays and was found to be irrelevant. Due to a sample *pH* below 1.1, the formation of significant amounts of hemicetals can also be excluded. It is conspicuous that the NMR resonances are broader in spectra obtained from buffered samples than in spectra from aqueous methanolic samples, this could be caused by self-association of the anthocyanidins in aqueous media. Such supramolecular aggregates are known to lead to reduced quantification due to the aggregates’ slower tumbling rate (stochastic rotational and diffusion motion in the solution). The longer correlation times of such aggregates lead to faster T2 (spin-spin) relaxation and can induce signal broadening [26].

The focus of the investigation was aqueous solvents because in vitro experiments are usually performed in buffer. However, some polyphenols have limited solubility in water; therefore, HPLC-DAD standard stock solutions are often prepared in aqueous alcohol, and quantification with q-NMR also requires relatively high concentrations. Therefore, aqueous methanol was also included in the study. Despite limited solubility, stacking and hydratisation in aqueous solvents might be problematic for quantification. If the molecules form more than simple van der Waals interactions with the solvent, as with hydrogen bonds or (de-)protonation equilibria, NMR signal intensities may be influenced due to the carry-over of water presaturation into the molecule (NOE).

Supramolecular stacking has an impact on the absorption spectra and the absorption coefficient and on the NMR resonances, too. However, for the UV/vis spectra, this effect is negligible due to high dilutions (1:50–1:400, 1:10,000 for CA to measure absorptions in the range of 0.1–1.4); for NMR, we observed (as expected) signal broadening and lowered intensities, and these effects were inversely proportional to the sample temperature during measurement. However, due to the limited amounts of substances and due to their tendency to degrade, we did not systematically acquire spectra at T(sample) > RT.

## 4. Materials and Methods

### 4.1. Materials, Solvents, and Reagents

(-)-Epicatechin (EC) (95.1% purity HPLC), 3-*O*-caffeoquinic acid (chlorogenic acid, CA) (99% titration with NaOH), 5-*O*-caffeoquinic acid (neochlorogenic acid, NCA) (99.5% HPLC), phlorizin dihydrate (PHL) (99% purity), *trans*-sinapinic acid (SIN) (99.1% HPLC, 100.1% titration), and *trans*-ferulic acid (FER) (99.8% purity HPLC; 99.8% titration) were stored at room temperature. 4-*O*-caffeoylquinic acid (cryptochlorogenic acid, CCA) (99.6% HPLC) and epigallocatechin gallate (EGCG) (99% HPLC) were stored at 4 °C and quercitin-3-*O*-glucoside (Q-3-glc) (91.4% HPLC), as well as resveratrol (RES) (100% HPLC) at −20 °C. These phenolic structures were obtained from Sigma Aldrich (Darmstadt, Germany).

(+)-Catechin (CAT) (99.5% HPLC-PDA) and 4,5-*O*-dicaffeoylquinic acid (DQA) (99.2% HPLC-PDA) were purchased from Phytolab GmbH & Co. KG (Germany) and stored at 4 °C. The procyanidins (PC) B1 (97.39%), B2 (96.72%), and C1 (97.41%), as well as *trans*-caffeic acid (CAF) (99.90% HPLC UV), *trans*-p-cumaric acid (COU) (99.76% HPLC-UV), and isorhamnetin-3-*O*-rutinoside (IRH-3-rut) (99.06% HPLC-UV), were also purchased from Phytolab and stored at −80 °C (PCs) and room temperature, respectively.

The anthocyanin-3-*O*-glucosides cyanidin-3-*O*-glucoside (CYD-3-glc) (99.66% HPLC), delphinidin-3-*O*-glucoside (DPD-3-glc) (98.11% HPLC), malvidin-3-*O*-glucoside (MLV-3-glc) (99.10% HPLC), pelargonidin-3-*O*-glucoside (PLG-3-glc) (98.95% HPLC), peonidin-3-*O*-glucoside (PEO-3-glc) (98.79% HPLC), and petunidin-3- *O*-glucoside (PET-3-glc) (98.27% HPLC) were obtained as chlorides from Phytolab GmbH & Co. KG (Germany) and stored at −80 °C.

Na_2_HPO_4_ and NaH_2_PO_4_∙H_2_O were obtained from Roth (Karlsruhe, Germany) to prepare 100 mM of phosphate buffer at *pH* 7.5. Sodium hydroxide and hydrochloric acid (Grüssing, Germany) were used to adjust the *pH* value. For NMR experiments, D_2_O and methanol-d_4_ were purchased from Eurisotop (Saarbrücken, Germany), and the methanol used to dilute the samples for UV spectroscopy was acquired from Fisher Scientific (Loughborough, UK). All reagents and solvents were of analytical grade and ultrapure water (ELGA PurLab flex, Veolia Waters, Celle, Germany) was used throughout.

### 4.2. Preparation of the Stock Solutions

Polyphenols were weighed using an AT 20 (Mettler Toledo; Gießen, Germany) balance. Anthocyanin stock solutions were prepared in ultrapure water containing 0.1% HCl, and all other phenolic structures were dissolved in 0.5 mL of methanol-d_4_ and subsequently mixed with 0.5 mL of D_2_O. All solvents were degassed and samples were stored at −20 °C. The compounds and mass concentrations (*γ*) determined by the balance and NMR are listed in Table 4.

### 4.3. Quantification Based on ^1^H-NMR

Absolute quantification of the polyphenols was performed in solution by quantitative nuclear magnetic resonance spectroscopy (qNMR) at the Chemical and Veterinary Investigation Office Karlsruhe (Chemisches Veterinär- und Untersuchungsamt, Karlsruhe, Germany). The measurement was carried out in methanol-d_4_/D_2_O (50/50, *v/v*) for the initial concentration and an appropriate dilution to check for concentration-dependent impacts. Initially, anthocyanins were quantified at two different concentrations (1.2–2.3 mM, diluted 1:4 and 1:6) in 0.2 M potassium chloride buffer adjusted to *pH* = 1 with 0.2 M of HCl and D_2_O. The *pH* value of the samples ranged between 1.05 and 1.10 after 1 h of equilibration. To investigate the systematic difference between the balance and qNMR, stock solutions of delphinidin-3-glucoside (D_2_O, 0.1% DCl) were diluted in potassium chloride buffer *pH* 1 and acidic methanol-d_4_/D_2_O (50/50, *v/v*, *pH* 1).

**Figure 5:**
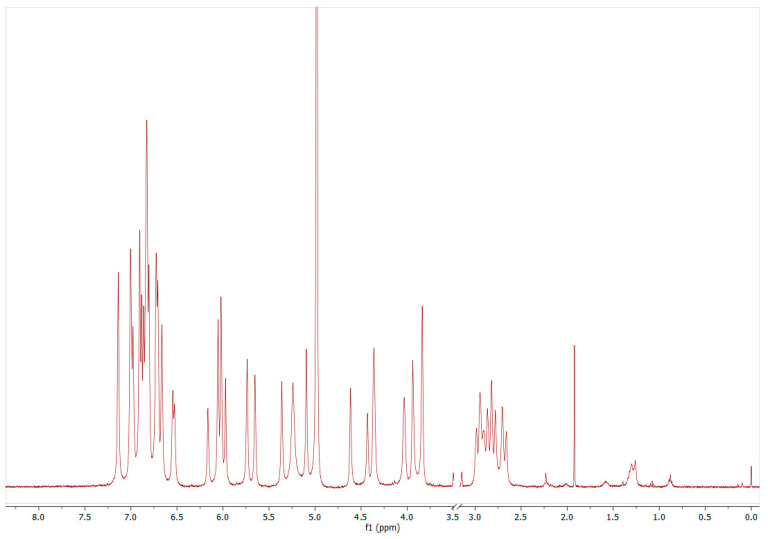
^1^H-NMR example spectrum of procyanidin-B2 (1868 mg/L) in methanol-d_4_/D_2_O (50/50, *v/v*). The signals in the range of 6.5–7.15 ppm (the six protons of Ring B and E) and 2.6–3.0 ppm (the two diastereomeric protons F4) were used for summary quantification (Figure 1, ^1^H-NMR spectra with signal assignments for all PP are provided in the Supplemental Material Figure S2, including references).

In general, the volume of 600 µL of the stock solutions was transferred into a 5 mm NMR tube and NMR spectra were recorded on a 400 MHz Bruker Avance (Bruker Biospin, Germany) equipped with a BBI 400S1 H-BB-D-05 Z probe and an automatic sample changer (Sample Xpress). Proton spectra were acquired using the pulse program noesygppr1d_d7 (1D NMR spectra) with presaturation of the water signal and an additional (fully passive) d7 delay limiting the presaturation irradiation to the d1 delay immediately before the excitation pulse. See Figure 5 as an example, for more spectra, see the Supplemental Material, Figure S2. To obtain an optimal and comparable excitation for all samples, the 90° pulse was calibrated for each sample using Bruker’s PULSECAL routine. With a time domain (TD) of 128 k, 128 scans with 4 dummy scans were acquired, using a spectral width (SW) of 20.56 ppm (8223 Hz), an acquisition time (AQ) of 7.97 s, and a receiver gain (RG) of 32. Delay 1 (D1) and delay 7 (D7) were set to 4.00 and 60.0 s, respectively. The sample temperature was set at 300 K (±0.1 K). All spectra were automatically phased and baseline-corrected. NMR spectra were analyzed using TopSpin version 4.06 (Bruker Biospin, Germany) and compound concentrations were determined using the PULCON principle (pulse length-based concentration determination) according to [14,39,40]. ^1^H-NMR spectra of Quantification Reference solutions (QuantRef, = external standards), containing known, purity-corrected concentrations of the certified reference substances lactic acid and citric acid (aqueous QR for anthocyanins) or diethyl phthalate and 1,2,4,5-tetrachloro-3-nitrobenzene (organic QR for nonanthocyanin phenolic structures) were used to calculate the ERETIC factor according to Equation (1).

(1) $$f_{ERETIC}=\frac{I_{\mathrm{Ref}}\times SW_{\mathrm{Ref}}\times M_{\mathrm{Ref}}}{SI_{\mathrm{Ref}}\times \gamma_{\mathrm{Ref},\;\mathrm{corr}}\times N_{H,\;\mathrm{Ref}}\times 1000}\;\left(\mathrm{in}\;\frac{a.u.\times \;\mathrm{ppm}\;\times \;L}{\mathrm{mmol}}\right)$$

where:

*I*_Ref_ = absolute integral of the reference signal;

*SW*_Ref_ = spectral width;

*M*_Ref_ = molar mass;

*SI*_Ref_ = number of data points of the processed reference spectrum;

*γ*_Ref,corr_ = mass concentration of reference substance, adjusted for purity;

*N*_H,Ref_ = number of protons per reference molecule giving this resonance.

The following factor was used to quantify the anthocyanins according to Equation (2).

(2) $$\gamma_{\mathrm{An}}=\frac{I_{\mathrm{An}}\times SW_{\mathrm{An}}\times M_{\mathrm{An}}}{SI_{\mathrm{An}}\times f_{ERETIC}\times N_{H,\;\mathrm{An}}\times f_{\mathrm{dil}}}\times \frac{P_{\mathrm{An}}}{P_{\mathrm{Ref}}}\times \frac{NS_{\mathrm{Ref}}}{NS_{\mathrm{An}}}\;\left(\mathrm{in}\;\frac{mg}{L}\right)$$

where:

*γ*_An_ = analyte mass concentration;

*I*_An_ = absolute integral of analyte in sample;

*SW*_An_ = spectral width;

*M*_An_ = molar weight of analyte;

*SI*_An_ = no. of data points of the processed analyte spectrum;

*f_ERETIC_* = mean value ERETIC factor from QuantRef;

*N*_H,An_ = number of protons per analyte molecule giving this resonance;

*f*_dil_ = dilution factor from analyte stock solution to measurement sample;

*P*_An_ = excitation pulse length used for the analyte sample (in µs);

*P*_Ref_ = excitation pulse length used for the QuantRef solution (in µs);

*NS*_Ref_ = number of recorded scans for the reference spectrum;

*NS*_An_ = number of recorded scans for the analyte spectrum.

Determination of the mass concentration *γ* was performed in duplicate and calculated as an average for the protons specified in Table 4. Signals for integration were selected having a low multiplicity and showing complete relaxation during the delay between the scans. The proton spectra are provided in the Supplementary Material.

### 4.4. Determination of the Absorption Coefficient

The absorptions were determined in duplicate by UV/Vis spectroscopy (Spectrostar Nano, BMG, Labtech, Ortenberg, Germany, UV-Cuvette semi micro-cuvette d = 1 cm, Helma Analytics, Muehlheim, Germany) after equilibration for, at minimum, three different dilutions. The absorption coefficients *ε* (in L·mol^−1^·cm^−1^) were calculated according to Equation (3) for each concentration and then expressed as mean ± standard deviation.

(3) $$\varepsilon =\frac{Abs\times f_{\mathrm{dil}}\times M_{an}}{\gamma_{an}\times l\times 1000}\;\left(\mathrm{in}\frac{L}{\mathrm{cm}\;\times \;\mathrm{mol}}\right)$$

*Abs* = absorption at *λ*_max_ or 280 nm;

*M_an_* = molar weight of the anthocyanin;

*γ_an_* = average mass concentration of the anthocyanin determined by q-NMR;

*l* = path length (1 cm);

*f*_dil_ = dilution factor;

1000 = conversion factor.

## 5. Conclusions

This article provides absorption coefficients for some phenolic structures in solvents generally used in experiments. The data also help to work with precise concentrations at low amounts during experiments and to save time and money. Commonly, it is recommended to use the absorption coefficients at *λ*_max_; however, due to equipment limitations, it might sometimes be required to use the coefficient obtained at 280 nm.

## Figures and Tables

**Table 1:** Absorption coefficients at *λ*_max_ (individual) for different phenolic compounds in methanol/water, water, and phosphate buffer *pH* 7.5 using concentrations determined by balance and q-NMR.

PP		Methanol/Water (50/50, *v/v*)							Water ^[a]^							Phosphate Buffer *pH* 7.5						Difference of *ε* between Calculation Based on q-NMR and Balance ^[b]^ (%)
		Balance			NMR				Balance			NMR				Balance			NMR			
	*λ*_max_ /nm	*ε* /(L·mol^−1^·cm^−1^)			*ε* /(L·mol^−1^·cm^−1^)			*λ*_max_ /nm	*ε* /(L·mol^−1^·cm^−1^)			*ε* /(L·mol^−1^·cm^−1^)			*λ*_max_ /nm	*ε* /(L·mol^−1^·cm^−1^)			*ε* /(L·mol^−1^·cm^−1^)			
GA	273	9507	±	436	9000	±	413	266	8021	±	166	7593	±	157	261	7406	±	288	7011	±	273	5.34
COU	309	18,279	±	1237	18,131	±	1227	290	17,867	±	301	17,722	±	298	287	16,216	±	187	16,084	±	186	0.81
CAF	322	15,458	±	590	14,792	±	565	315	14,606	±	601	13,976	±	575	312	12,073	±	266	11,553	±	255	4.31
FER	320	15,573	±	555	16,203	±	580	314	14,365	±	391	14,948	±	407	310	13,738	±	544	13,662	±	541	4.05
SIN	320	16,013	±	926	16,703	±	966	313	16,169	±	386	16,866	±	402	307	9743	±	510	10,163	±	532	4.31
CA	329	18,295	±	1435	18,091	±	1419	325	18,822	±	453	18,575	±	447	326	17,758	±	577	17,560	±	571	1.12
CCA	329	18,106	±	391	17,842	±	386	326	18,177	±	275	17,912	±	271	327	16,145	±	220	15,910	±	217	1.46
NCA	329	18,655	±	1084	18,323	±	1064	325	17,682	±	68	17,367	±	67	327	20,309	±	534	19,947	±	525	1.78
DCQ	330	34,027	±	1672	34,315	±	1686	325	30,331	±	612	30,587	±	617	328	29,988	±	1422	30,234	±	1431	0.85
CAT	280	4175	±	160	4047	±	155	280	3770	±	71	3655	±	69	280	3442	±	191	3337	±	185	3.06
EC	280	3981	±	73	3720	±	68	279	3771	±	83	3524	±	77	279	3714	±	157	3470	±	147	6.56
PC B1	281	7364	±	78	7534	±	80	280	7066	±	60	7229	±	62	280	6161	±	699 ^[c,e]^	6302	±	715 ^[c,e]^	2.30
PC B2	281	7496	±	223	7959	±	237	280	6810	±	83	7231	±	88	280	6698	±	189 ^[c]^	7112	±	201 ^[c]^	6.19
								280	7144	±	270 ^[d]^				280	7026	±	198 ^[d]^				
PC C1	281	11,542	±	802	15,397	±	1070	280	10,432	±	392	13,917	±	524	280	9783	±	533 ^[c]^	13,051	±	711 ^[c]^	33.40
EGCG	277	10,735	±	819	11,958	±	912	275	10,438	±	190	11,628	±	211	277	9525	±	255	10,610	±	284	11.39
IRH-3-rut	257	22,001	±	744	22,381	±	756	256	18,925	±	499	19,252	±	508	270	19,760	±	844	20,101	±	859	1.73
	357	19,075	±	646	19,404	±	657	353	15,850	±	415	16,123	±	422	362	14,216	±	618	14,461	±	629	1.73
Q-3-glc	258	19,568	±	938 ^[f]^	25,053	±	1201	257	17,629	±	955 ^[f]^	22,570	±	1223	269	17,545	±	368 ^[f]^	22,464	±	471	28.03
	358	16,317	±	731 ^[f]^	21,515	±	964	352	13,915	±	782 ^[f]^	18,349	±	1031	365	12,009	±	215 ^[f]^	15,836	±	283	31.86
RES	307	28,195	±	77	28,348	±	77	307	26,351	±	477	26,494	±	480	307	28,150	±	488 ^[c]^	28,303	±	491 ^[c]^	0.54
								307	25,455	±	874 ^[d]^				307	28,340	±	492 ^[d]^				
PHL	287	15,585	±	267	15,139	±	260	286	14,986	±	100	14,557	±	97	325	18,164	±	179	17,643	±	176	2.86

^[a]^ The respective *pH* values are provided in Table A1; ^[b]^ (*ε*_q-NMR-_ε_balance_)/*ε*_balance_ × 100%; ^[c]^ the concentration of the solution was determined by UV spectroscopy with the absorption coefficient obtained in water; ^[d]^ the value is calculated with a second sample based on weight. ^[e]^ based on the values determined for PC B2, the values seem to be underestimated, ^[f]^ guaranteed purity is less than 90% (HPLC); therefore, the absorption coefficient might be underestimated. Due to unknown exact purity, the calculation is based on an estimated purity of 100%.

**Table 2:** Absorption coefficients at 280 nm for different phenolic compounds in methanol/water, water, and phosphate buffer *pH* 7.5 using concentrations determined by balance and q-NMR.

PP	Methanol/Water (50/50, *v/v*)						Water ^[a]^						Phosphate Buffer *pH* 7.5						Difference of ε between Calculation Based on q-NMR and Balance ^[b]^ (%)
	Balance			NMR			Balance			NMR			Balance			NMR			
	ε/(L·mol^−1^·cm^−1^)			ε/(L·mol^−1^·cm^−1^)			ε/(L·mol^−1^·cm^−1^)			ε/(L·mol^−1^·cm^−1^)			ε/(L·mol^−1^·cm^−1^)			ε/(L·mol^−1^·cm^−1^)			
GA	8635	±	521	8174	±	493	5901	±	595	5586	±	563	3703	±	98	3505	±	92	5.34
COU	14,035	±	471	13,921	±	467	15,982	±	548	15,852	±	544	15,470	±	162	15,344	±	160	0.81
CAF	10,491	±	564	10,039	±	540	12,376	±	550	11,843	±	526	11,722	±	247	11,217	±	237	4.31
FER	10,310	±	408	10,728	±	425	11,786	±	363	12,264	±	378	13,041	±	511	13,570	±	532	4.06
SIN	5894	±	365	6148	±	381	7985	±	718	8329	±	749	6042	±	310	6303	±	323	4.31
CA	8002	±	500	7913	±	494	10,119	±	264	9987	±	260	9231	±	262	9128	±	259	1.12
CCA	7893	±	189	7778	±	186	9176	±	138	9042	±	136	7942	±	127	7826	±	125	1.46
NCA	8544	±	477	8392	±	468	9189	±	69	9026	±	68	10,292	±	239	10,108	±	235	1.78
DCQ	14,961	±	669	15,087	±	675	15,644	±	341	15,776	±	343	15,188	±	562	15,317	±	567	0.85
CAT	4175	±	160	4047	±	155	3770	±	71	3655	±	69	3442	±	191	3337	±	185	3.06
EC	3981	±	73	3720	±	68	3754	±	83	3508	±	78	3702	±	159	3459	±	149	6.56
PC B1	7346	±	79	7515	±	81	7066	±	60	7229	±	62	6161	±	699 ^[c,e]^	6302	±	715 ^[c,e]^	2.30
PC B2	7482	±	227	7945	±	241	6810	±	83	7231	±	88	6698	±	189 ^[c]^	7112	±	201 ^[c]^	6.19
							7144	±	270 ^[d]^				7026	±	198 ^[d]^				
PC C1	11,518	±	802	15,366	±	1070	10,432	±	392	13,917	±	524	9783	±	533 ^[c]^	13,051	±	711 ^[c]^	33.40
EGCG	10,544	±	806	11,745	±	898	9970	±	197	11,106	±	219	9319	±	205	10,381	±	229	11.39
IRH-3-rut	8958	±	313	9112	±	319	8190	±	155	8331	±	158	13,588	±	587	13,823	±	597	1.73
Q-3-glc	7898	±	296 ^[f]^	10,112	±	379	7474	±	452 ^[f]^	9569	±	579	11,166	±	201 ^[f]^	14,296	±	257	28.03
RES	13,731	±	150	13,805	±	151	13,483	±	217	13,556	±	218	13,889	±	184 ^[c]^	13,964	±	185 ^[c]^	0.54
							12,778	±	658 ^[d]^				13,983	±	185 ^[d]^				
PHL	14,187	±	229	13,781	±	223	13,940	±	83	13,541	±	81	8318	±	185	8080	±	180	2.86

^[a]^ The respective *pH* value is provided in Table A1; ^[b]^ (*ε*_q-NMR-_ε_balance_)/*ε*_balance_ × 100%; ^[c]^ the concentration of the solution was determined by UV spectroscopy with the absorption coefficient obtained in water; ^[d]^ the value is calculated with a second sample based on weight. ^[e]^ based on the values determined for PC B2, the values seem to be underestimated. ^[f]^ guaranteed purity is less than 90% (HPLC); therefore, the absorption coefficient might be underestimated. Due to unknown exact purity, the calculation is based on an estimated purity of 100%.

**Table 3:** Absorption coefficients for different anthocyanins in potassium chloride buffer at *pH* 1 at 520 nm and *λ*_max_ using concentrations determined by balance.

ACY	*ε*_520nm_			*λ*_max_	*ε_λ_*_max_			*ε* According to [10]
	/(L·mol^−1^·cm^−1^)			/nm	/(L·mol^−1^·cm^−1^)			
PEL-3-glc	15,849	±	2070	497	21,843	±	2825	27,300
CYD-3-glc	25,526	±	428	510	26,953	±	464	26,900
DPD-3-glc	26,935	±	680	516	27,087	±	671	
PET-3-glc	26,821	±	1386	516	26,892	±	1353	
PEO-3-glc	23,926	±	898	510	25,141	±	931	
MLV-3-glc	27,911	±	437	518	27,923	±	443	28,000

**Table 4:** Mass concentration *γ* of the phenolic solutions based on the weights and determined with q-NMR at two different solutions.

PP	Quantification							Difference between Balance/NMR (in %)	Literature for Signal Assignment
	by Balance	by q-NMR spectroscopy							
	*γ* /(mg/L)	Protons Used for Quantification ^[a]^	*γ*_c_ /(mg/L)	*γ*_D_ /(mg/L) ^[b]^	Average /(mg/L)				
GA	2368	H 2,6	2573	405	2502	±	36	5.6	[27]
COU	1158	H_b_; H 2,6; H 3,5; H_a_	1219	186	1168	±	26	0.8	[28]
CAF	1088	H_b_; H 2; H 6; H 5; H_a_	1164	185	1137	±	14	4.5	[28]
FER	1244	H_b_; H 2; H 6; H 5; H_a_; H 7	1251	190	1196	±	28	3.9	[29]
SIN	2094	H_b_; H 2,6; H_a_; H 7,8	2041	329	2008	±	17	4.1	[30]
CA	26,990	H_b_; H 2′; H 6′; H 5′; H_a_	27,451	4523	27,295	±	78	1.1	[31]
CCA	2976	H_b_; H 2′; H 6′; H 5′; H_a_	3016	504	3020	±	2	1.5	[32]
NCA	6096	H_b_; H 2′; H 6′; H 5′; H_a_	6197	1036	6207	±	5	1.8	[31]
DCQ	3100	H_b′b″_; H 2′2″; H 6′6″; H 5′5″; H_a′a″_	3088	510	3074	±	7	0.8	[33]
CAT	1502	H 2′,5′; H6′; H 4_eq/ax_	1550	258	1549	±	1	3.1	[34]
EC	9362 ^[a]^	H 2′; H 5′,6′; H 4_eq/ax_	10,025	1669	10,020	±	3	7.0	[34]
PC B1	3954	H B 2′,5′,6′ + E 2′,5′,6; H F4_eq/ax_	3865					2.3	[22]
PC B2	5114	H B 2′,5′,6′ + E 2′,5′,6; H F4_eq/ax_	4816					5.8	[22]
PC C1	2492	H B 2′,5′,6′ + E 2′,5′,6 + H 2′,5′,6′; H I4_eq/ax_	1868					25.0	[23]
EGCG	1144	H 2″6″; H 2′6′; H 4_eq/ax_	1036	509 ^[c]^	1027	±	5	10.2	[27]
IRH-3-rut	1002	H 2′; H 6′; H 5′; H 13″	998	324 ^[d]^	985	±	7	1.7	[35]
Q-3-glc	2576 ^[d]^	H 2′; H 6′; H 5′; H 8; H 6	1954	345	2012	±	29	21.9	[36]
RES	1112 ^[d,e]^	H 2′,6′; H_b_; H 2,6; H 4	1144	178	1106	±	19	0.5	[37]
PHL	4679	H 2,6; H 3,5; H 3′; Hb	4817	798	4803	±	7	2.6	[38]

^[a]^ for further information, see spectra provided in the Supplementary Material Figure S2, ^[b]^ dilution factor 6, ^[c]^ dilution factor 2, ^[d]^ pre-dissolved in 0.5 µL of DMSO-d_6_; ^[e]^ purity declaration was >90% (HPLC). The difference between balance and qNMR is reduced to 15% assuming a purity of 90% for the Q-3-glc standard compound.

## Data Availability

Not applicable.

## Supplementary Materials

Table S1: Absorption coefficients of anthocyanidin-3-glucosides calculated by mass concentration γ determined by balance and q-NMR in aqueous buffer at pH 1. Figure S1: Proton spectra recorded with a 400 MHz spectrometer and used for quantification, including signal assignment based on the literature of delphinidin-3-O-glucoside in buffer. Table S2: Mass concentration γ determined by q-NMR in acidic methanol/water (50/50, *v/v*) and potassium chloride buffer pH 1. Figure S2: Proton spectra recorded with a 400 MHz spectrometer and used for quantification, including signal assignment based on the literature and own 2D NMR spectra.

## Sample Availability

Not available.

## Appendix A

**Table A1:** *pH* values for aqueous solution of the phenolic compounds in the specified concentration range.

PP	*c*/µM			*pH*		
GA	35	−	139	4.62	±	0.21
COU	18	−	71	4.92	±	0.24
CAF	15	−	60	4.96	±	0.18
FER	16	−	64	4.92	±	0.22
SIN	23	−	93	5.16	±	0.20
CA	8	−	38	5.00	±	0.36
CCA	21	−	84	4.62	±	0.30
NCA	17	−	86	5.64	±	0.08
DCQ	6	−	30	5.26	±	0.16
CAT	26	−	103	5.94	±	0.17
ECAT	81	−	323	6.10	±	0.11
PC B1	27	−	220	6.07	±	0.34
PC B2	25	−	197	6.13	±	0.08
PC C2	26	−	102	5.97	±	0.26
EGCG	6	−	50	6.02	±	0.01
IRH-3-rut	16	−	64	6.62	±	0.07
Q-3-glc	14	−	55	6.02	±	0.22
RES	9	−	37	6.33	±	0.44
PHL	27	−	107	6.01	±	0.03
