# Supplementary material for: Absorption Coefficients of Phenolic Structures in Different Solvents Routinely Used for Experiments
Source: Molecules. 2021 Jul 31;26(15):4656. doi: 10.3390/molecules26154656 (PMC8348453; doi:10.3390/molecules26154656)
Supplement: Supplementary file 1 [file molecules-26-04656-s001.zip › molecules-1302134-supplementaty.pdf]

Supplemental material

Table S1. Absorption coefficients of anthocyanidin-3-glucosides calculated by mass concentration γ determined by balance and q-NMR in aqueous buffer at pH 1.

ACY	λ = 520 nm		λ max			Difference of ε between calculation based on q-NMR and balance [b] [%]	ε according to [10]
	balance	NMR		balance	NMR		
	ε/ (L · mol^{-1} ·cm^{-1})	ε/ (L · mol^{-1} ·cm^{-1})	λ_{max} [nm]	ε/ (L · mol^{-1} ·cm^{-1})	ε/ (L · mol^{-1} ·cm^{-1})		
PEL-3-glc	15849 ± 2070	20317 ± 423	497	21843 ± 2825	28006 ± 774	128	27300
CYD-3-glc	25526 ± 428	29619 ± 167	510	26953 ± 464	31275 ± 270	116	26900
DPD-3-glc	26935 ± 680	34070 ± 580	516	27087 ± 671	34263 ± 567	127	
PET-3-glc	26821 ± 1386	37438 ± 140	516	26892 ± 1353	37540 ± 192	140	
PEO-3-glc	23926 ± 898	29451 ± 185	510	25141 ± 931	30947 ± 210	123	
MLV-3-glc	27911 ± 437	33140 ± 251	518	27923 ± 443	33154 ± 257	119	28000

Figure S1. Proton spectra recorded at a 400 MHz Spectrometer of delphinidin-3-O-glucoside in buffer pH 1

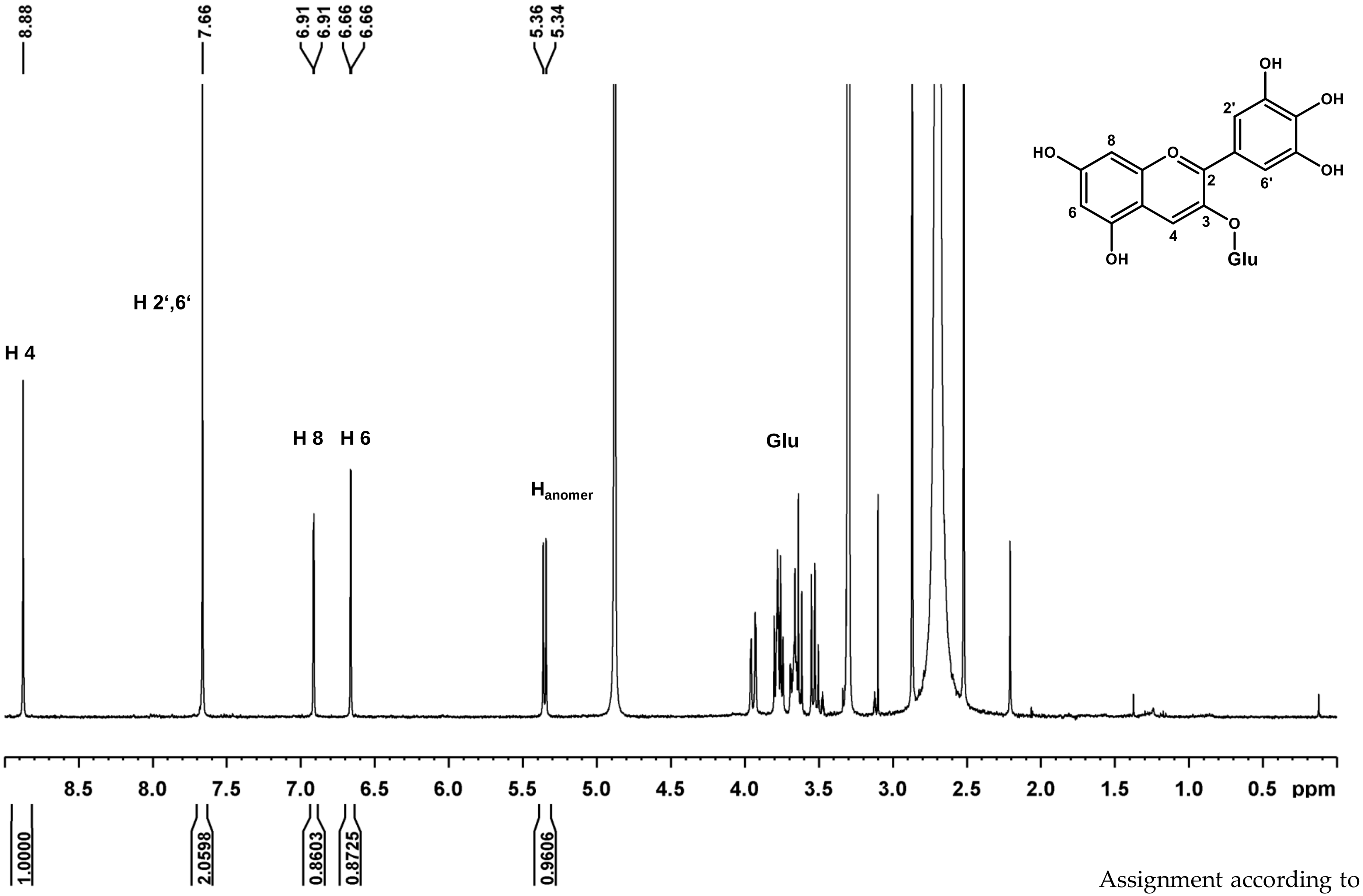

methanol-d_4/ water

Assignment according to Fernandes et al. (2015). New Journal of Chemistry, 39, 2602.

Supplemental material

Table S2. Mass concentration γ determined by q-NMR in acidic methanol/water (50/50, v/v) and potassium chloride buffer pH 1.

	Quantification					
	by balance	by q-NMR spectroscopy				
	γ/ (mg/L)	protons for quantification	$\gamma_{methanol/water}$ /(mg/L)	γ_{buffer} /(mg/L)[b]	Difference between balance/NMR [%] methanol/water	buffer
Dpd-3-glc 1	1522	H 4; H 2',6'; H 8; H6; H_{anomer}	1408	1120	93	74
Dpd-3-glc 2	979	H 4; H 2',6'; H 8; H6; H_{anomer}	885	733	90	75
Dpd-3-glc 1	1522	H 4; H 2',6'	1520	1145	100	75
Dpd-3-glc 2	979	H 4; H 2',6'	937	778	96	79

Supplemental material

Figure S2. Proton spectra recorded at a 400 MHz Spectrometer and used for quantification including signal assignment based on literature and own 2D NMR spectra in methanol-d_4/ D_2O

gallic acid

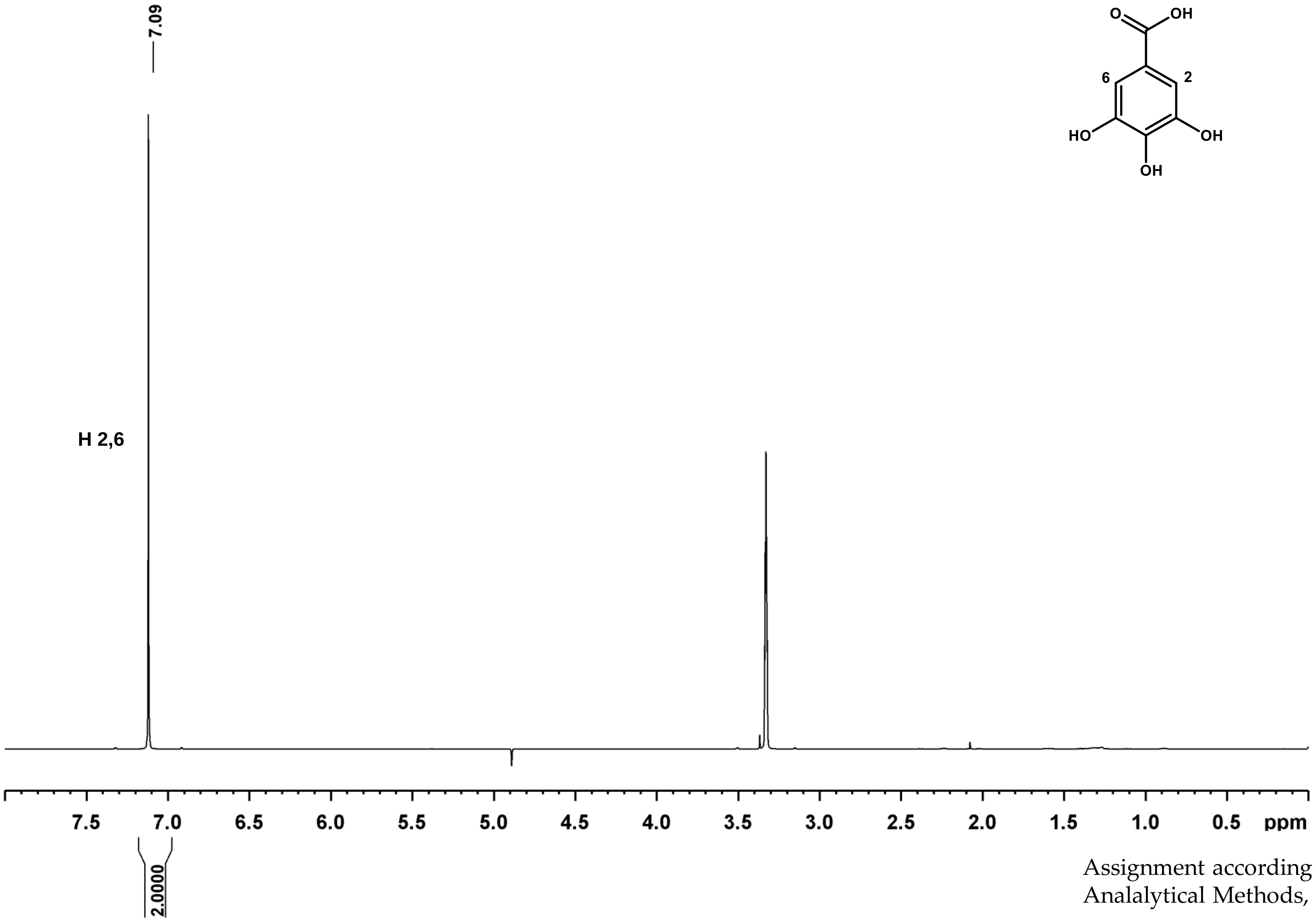

Assignment according to Yuan et al. (2014). Analalytical Methods, 6, 907-914.

trans-ferulic acid

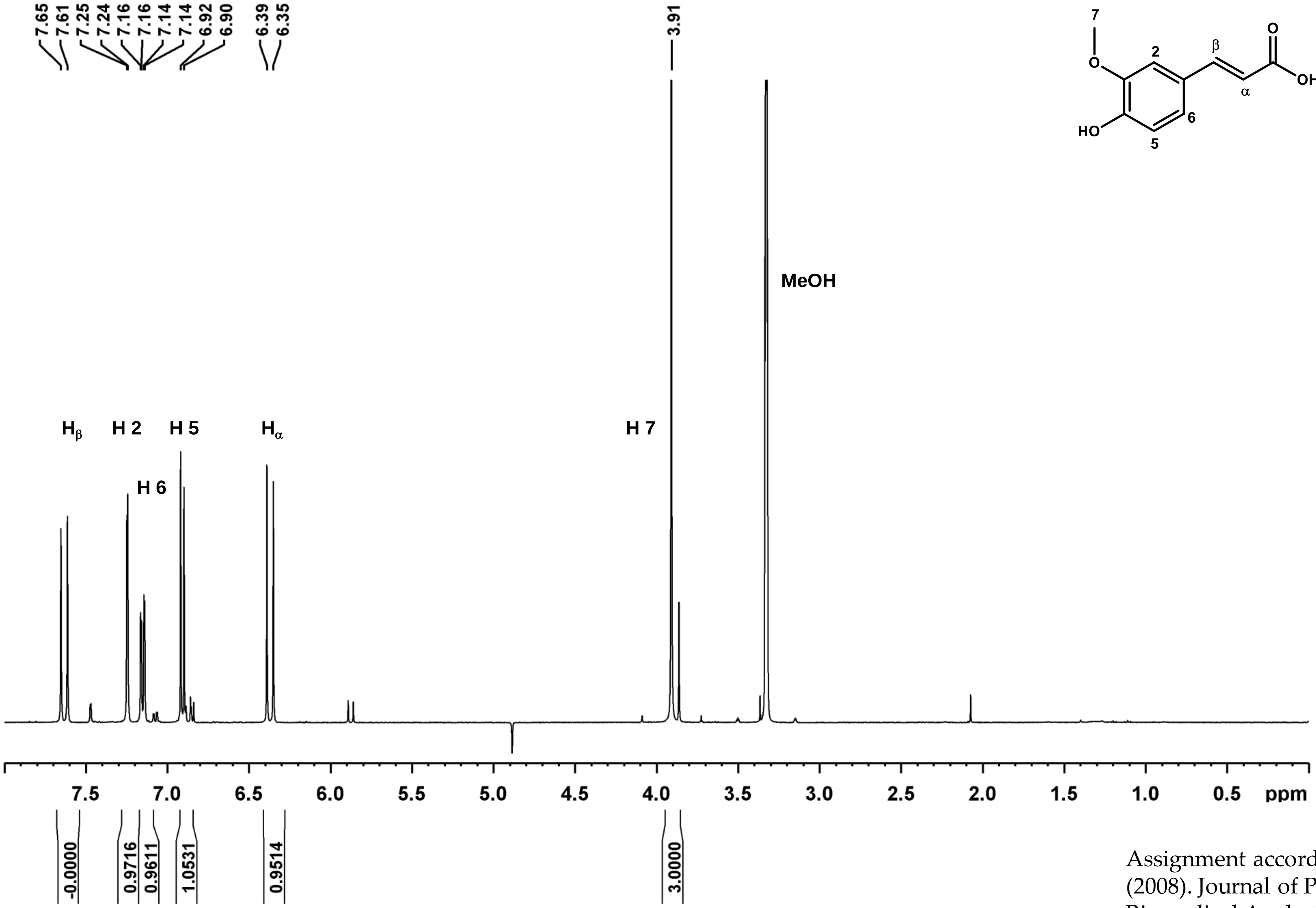

Assignment according to Anselmi et al. (2008). Journal of Pharmaceutical and Biomedical Analysis 46, 4, 645-652.

trans-caffeic acid

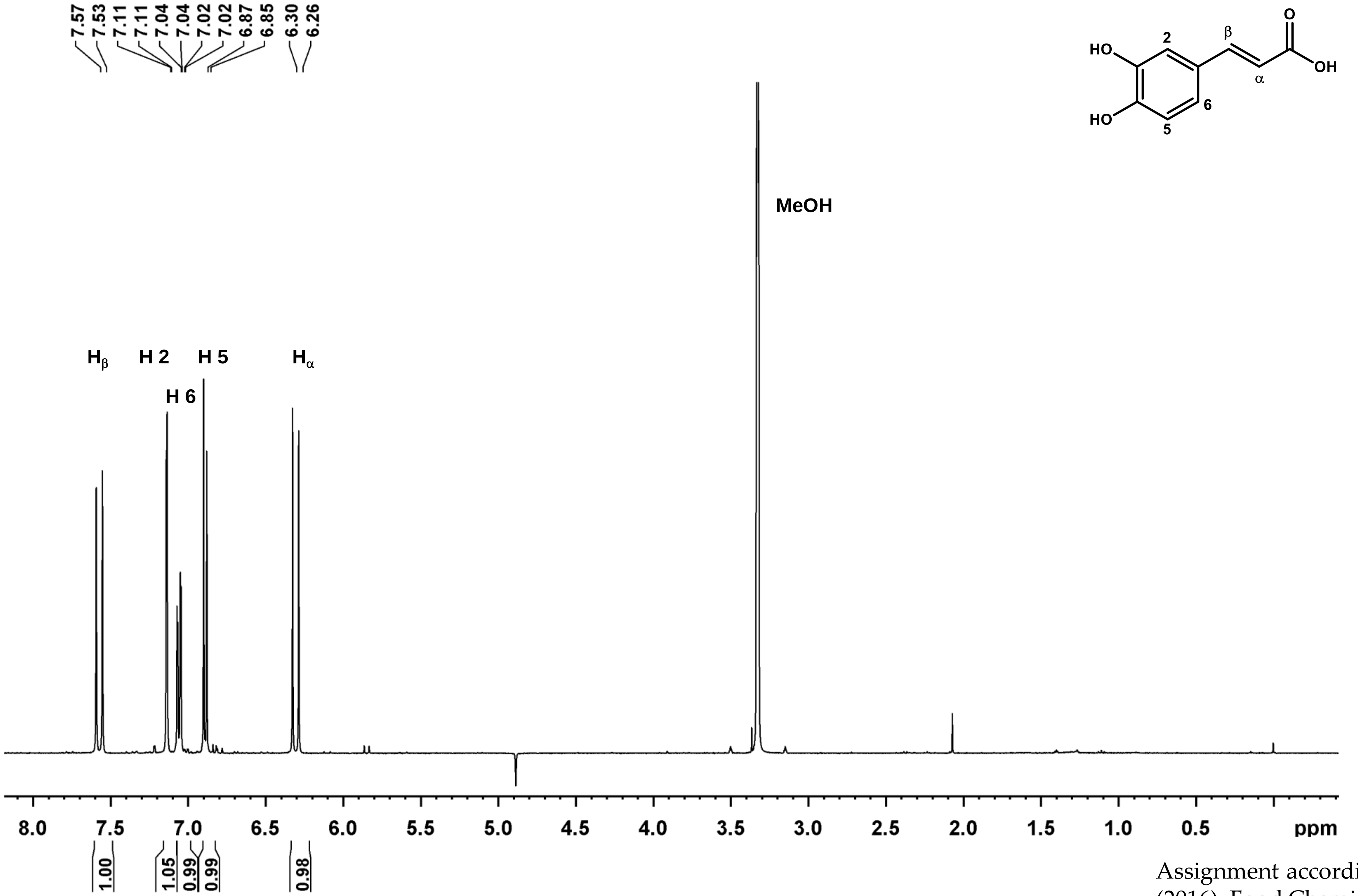

Assignment according to Forino et al. (2016). Food Chemistry, 194, 1254-1259.

trans-coumaric acid

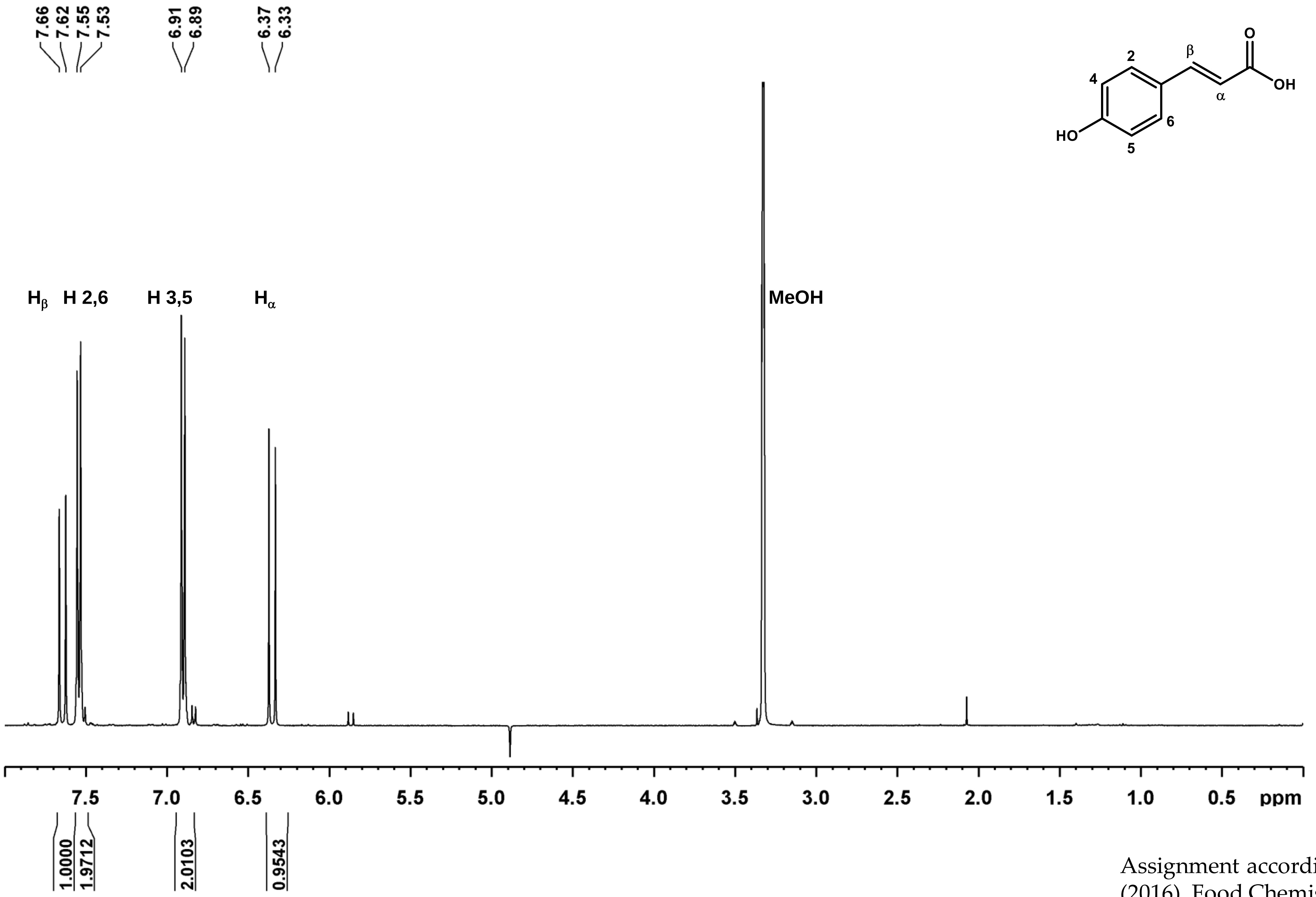

Assignment according to Forino et al. (2016). Food Chemistry, 194, 1254-1259.

trans-sinapinic acid

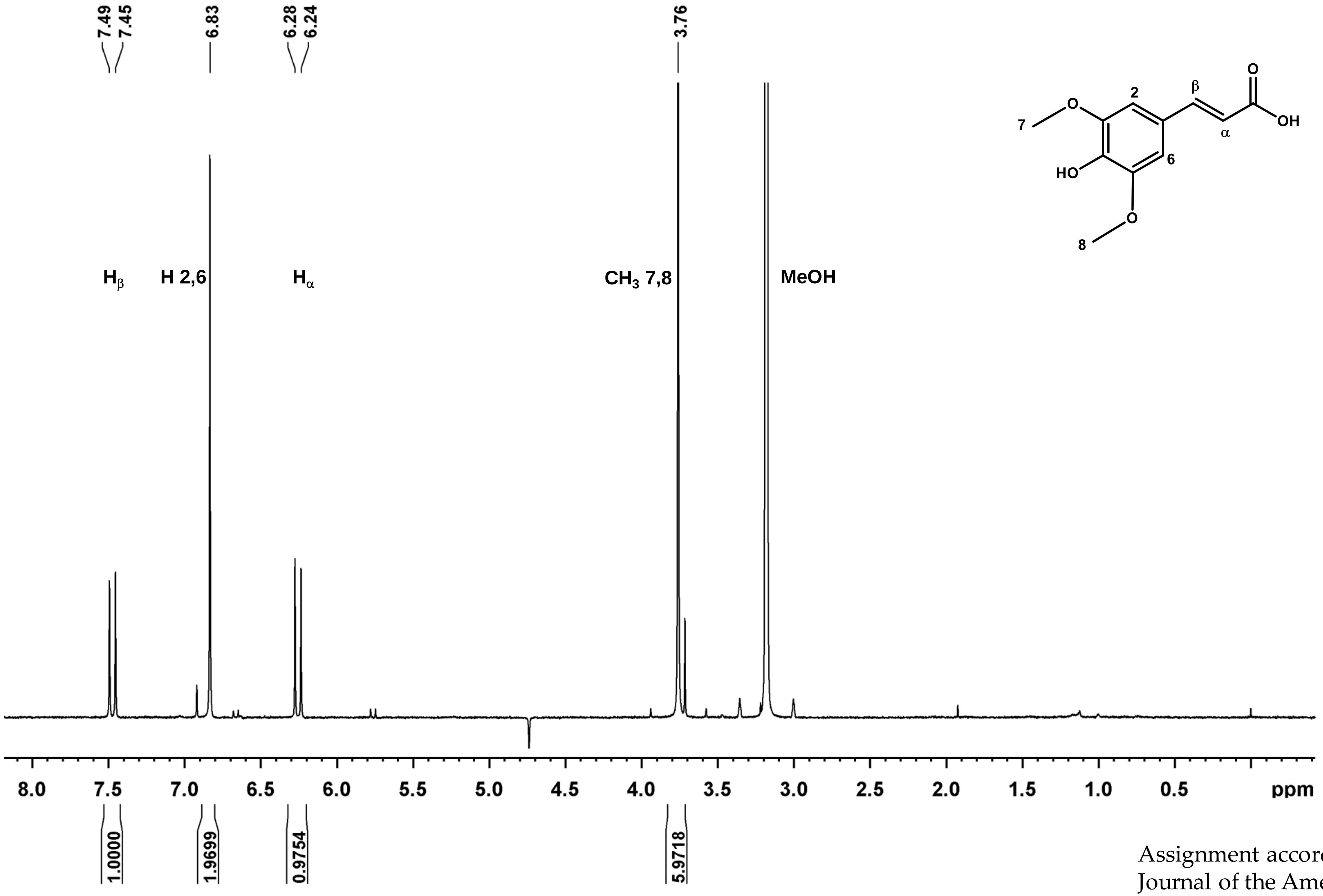

Assignment according to Cai et al. (1999) Journal of the American Oil Chemists, 76, 4, 433-441.

chlorogenic acid

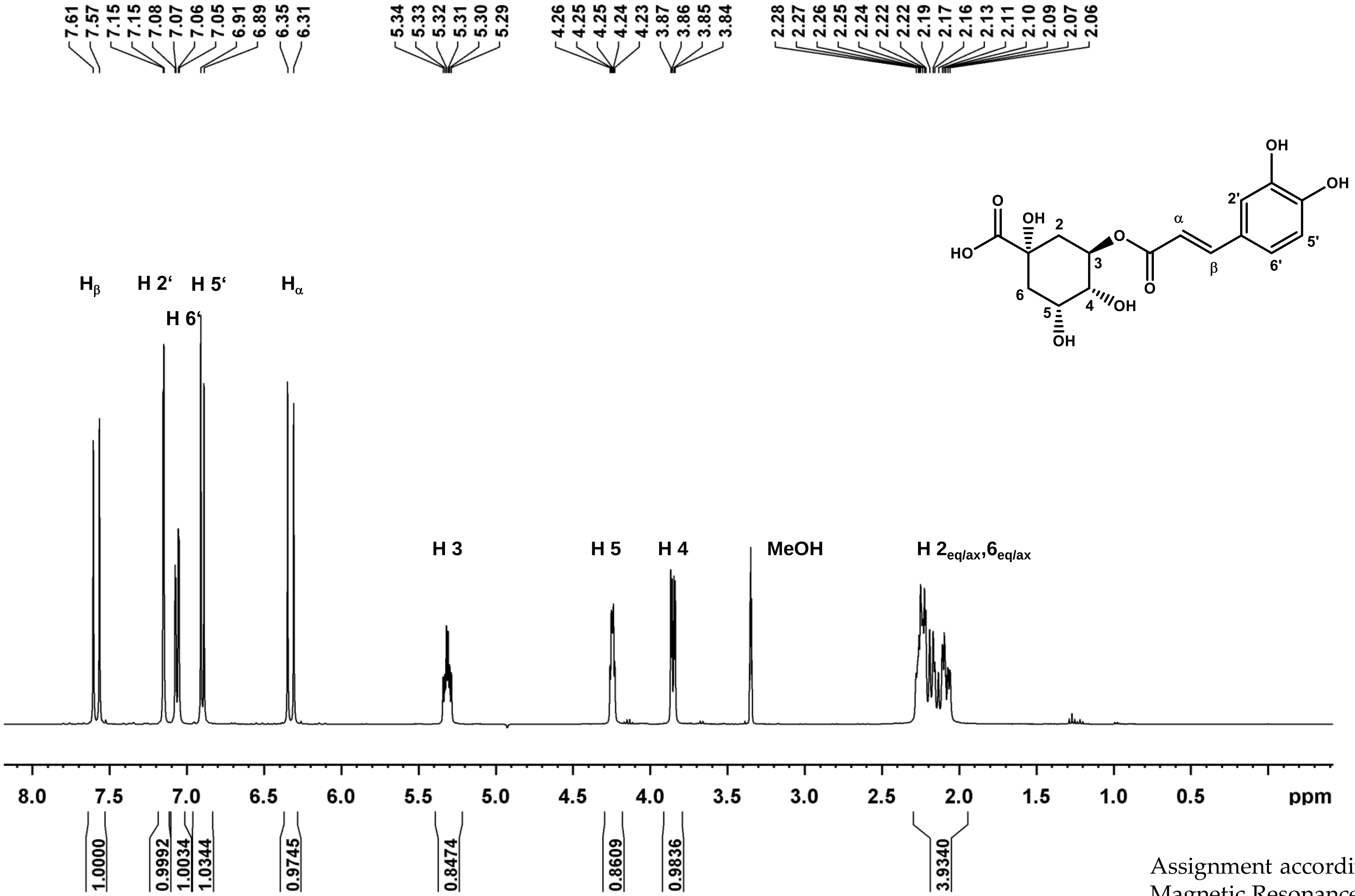

Assignment according to Pauli et al. (1999). Magnetic Resonance in Chemistry. Chem. 37, 11, 827-836.

cryptochlorogenic acid

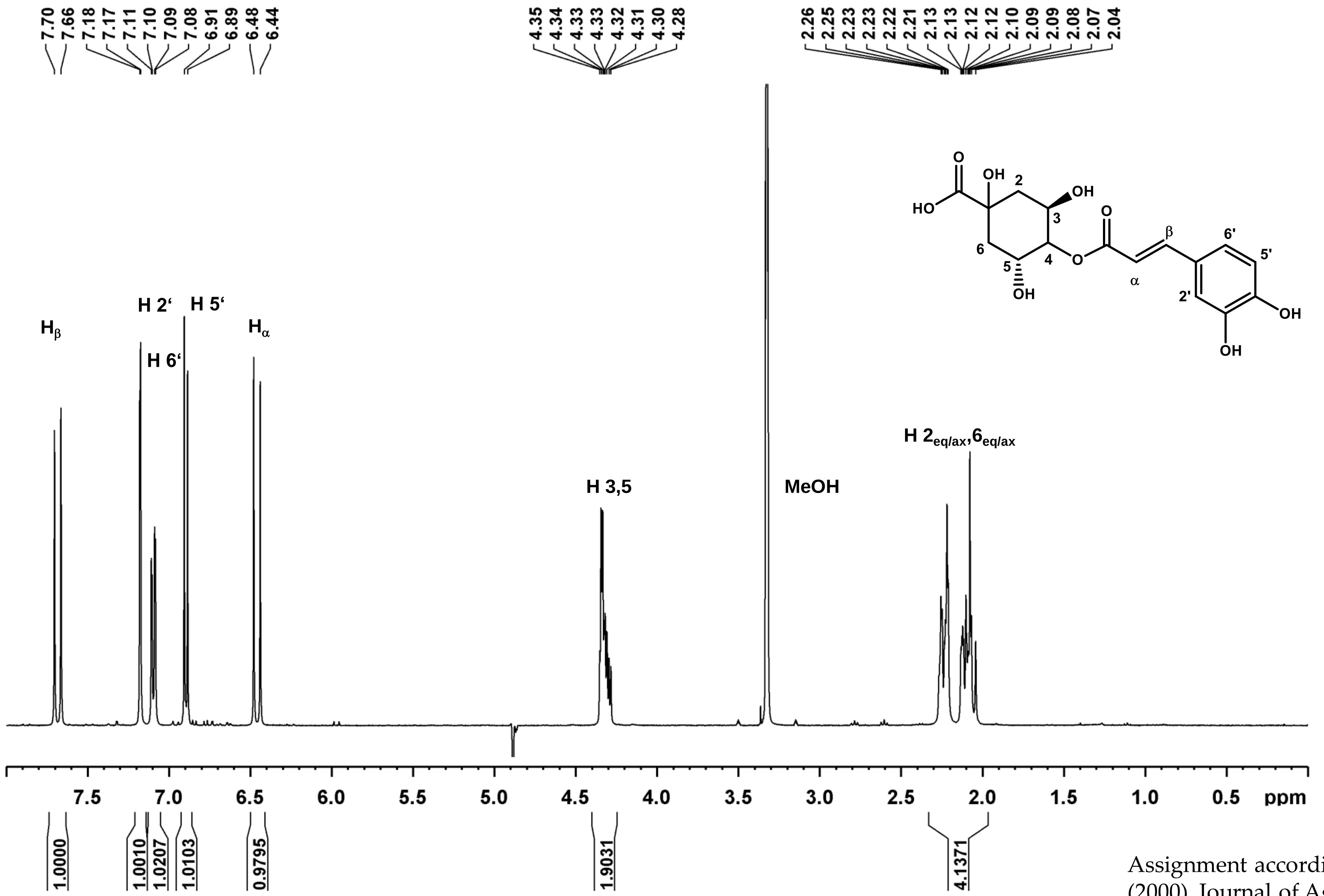

Assignment according to Nakatani et al. (2000). Journal of Agricultural and Food Chemistry, 48, 5512-5516.

neochlorogenic acid

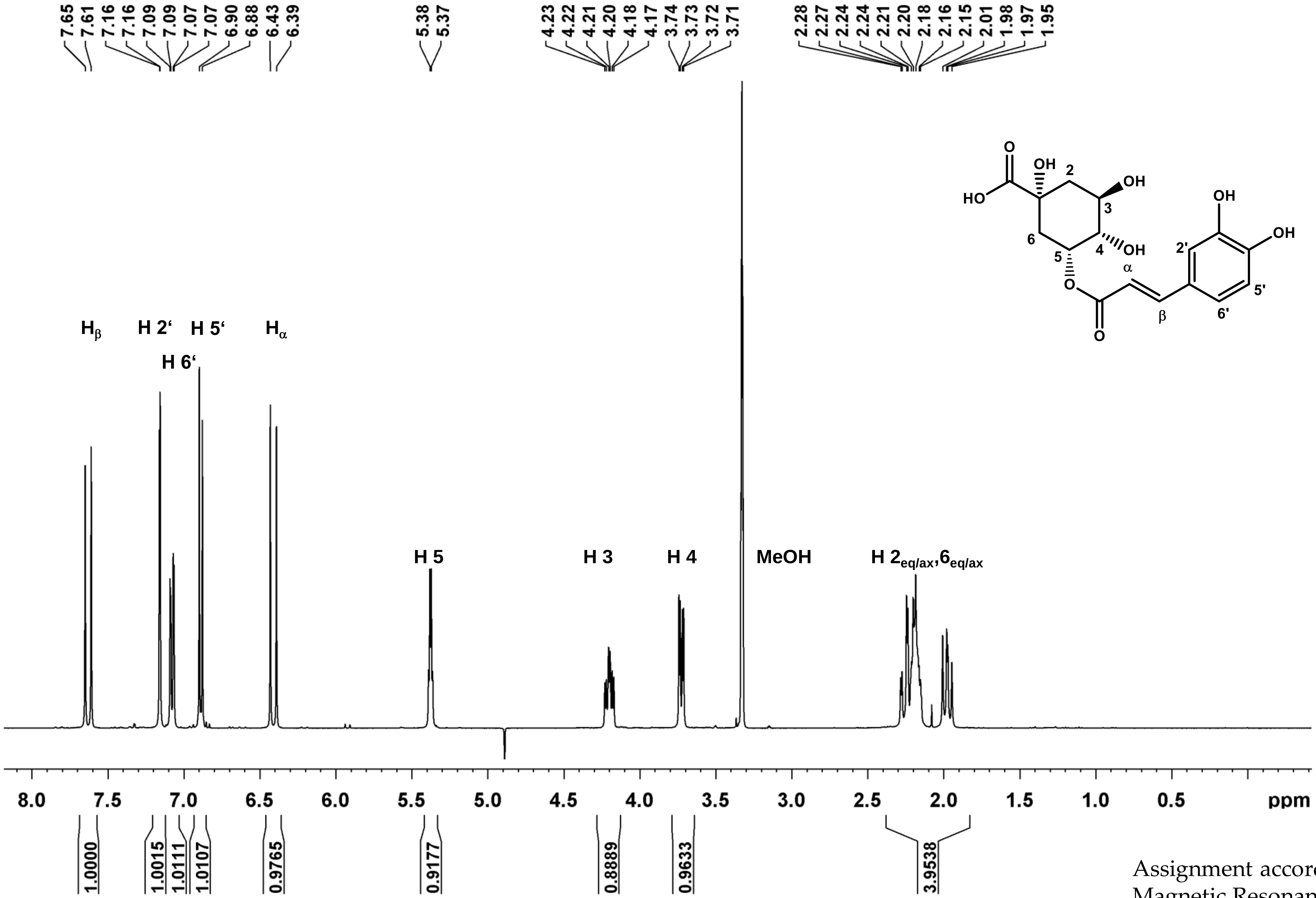

Assignment according to Pauli et al. (1999). Magnetic Resonance in Chemistry. Chem. 37, 11, 827-836.

4,5-dicaffeoylquinic acid

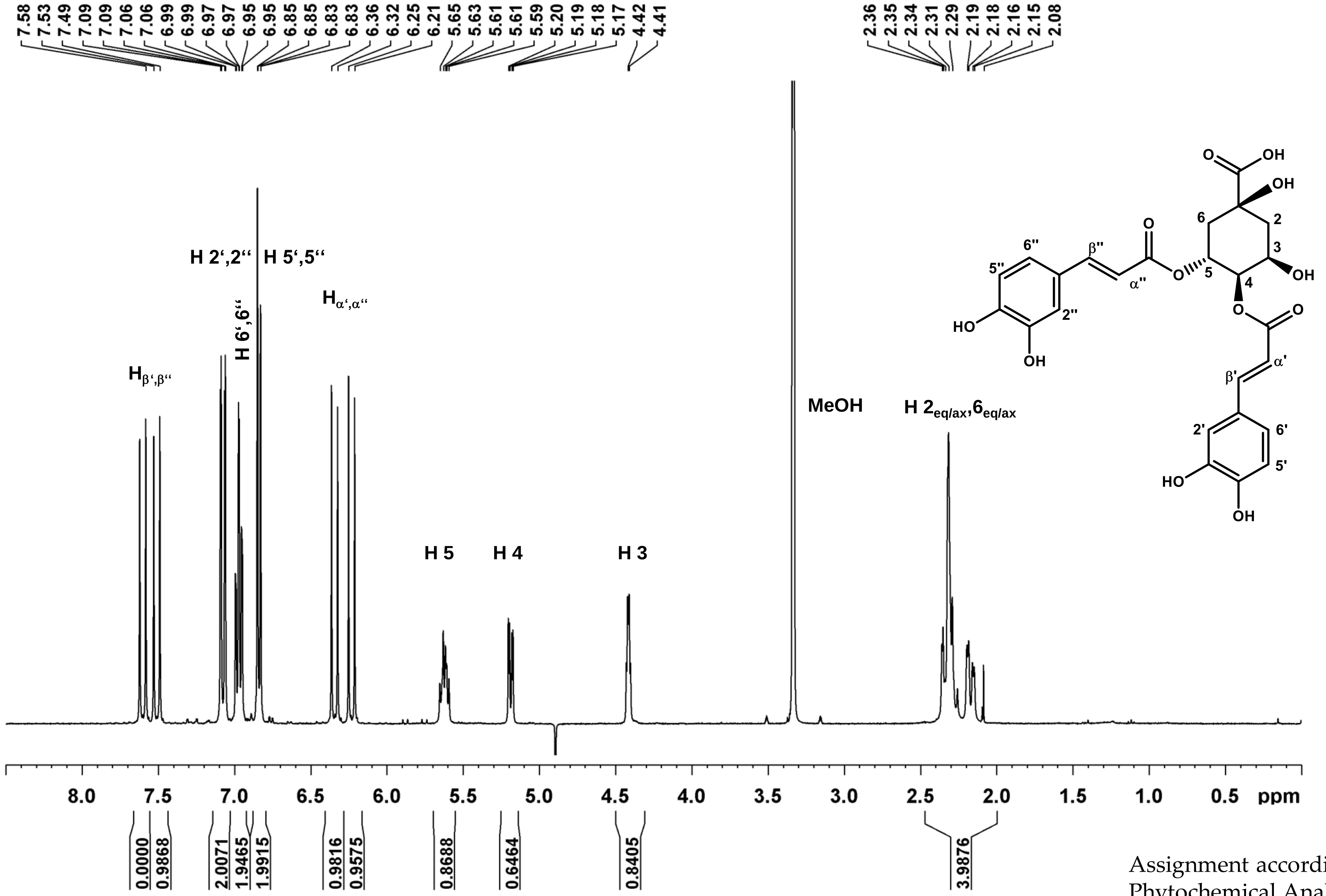

Assignment according to Wu et al. (2007). Phytochemical Analysis, 18, 5, 401-410.

(+)-catechin

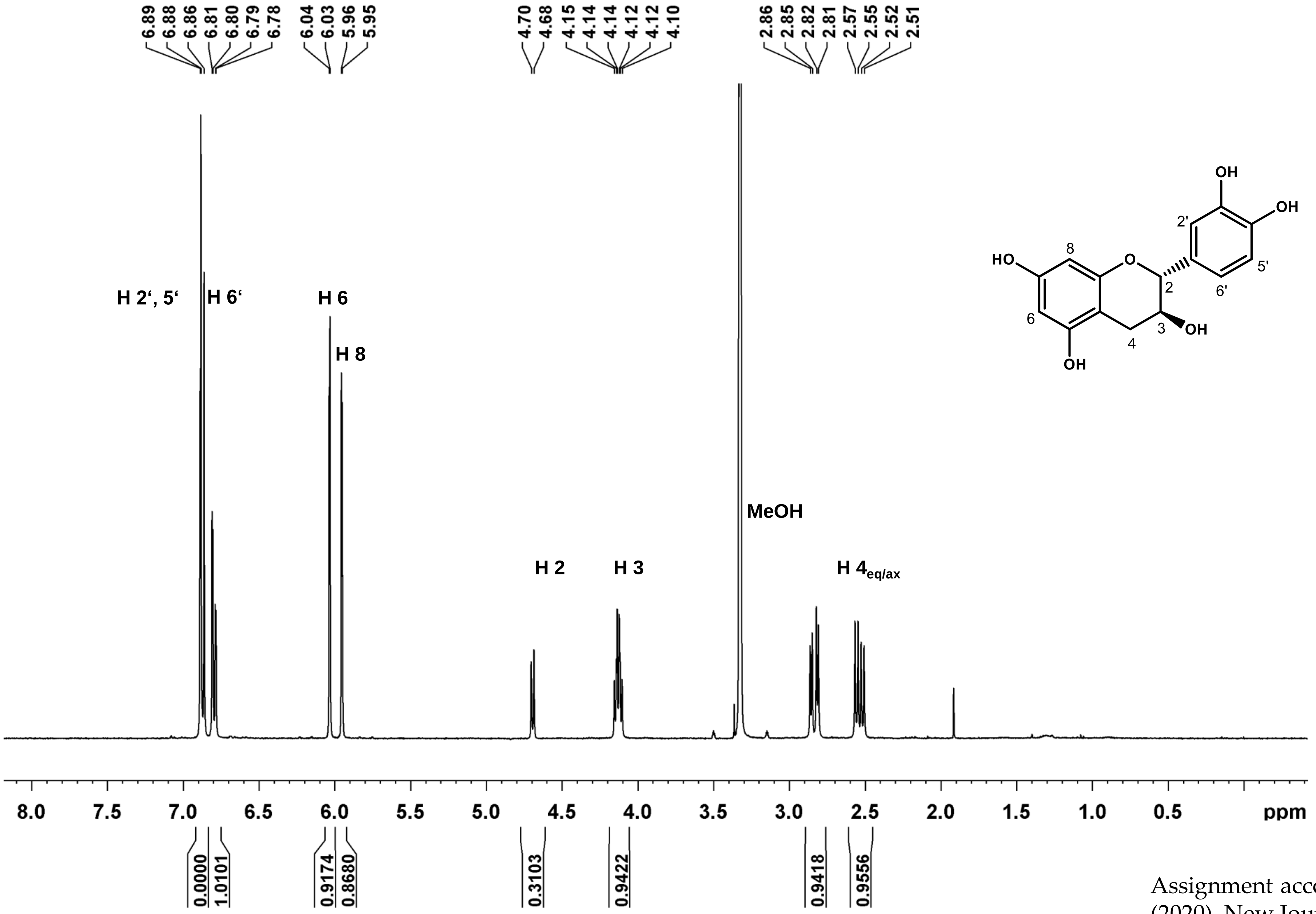

Assignment according to da Silva et al. (2020). New Journal of Chemistry, 44, 17391-17404.

(-)-epicatechin

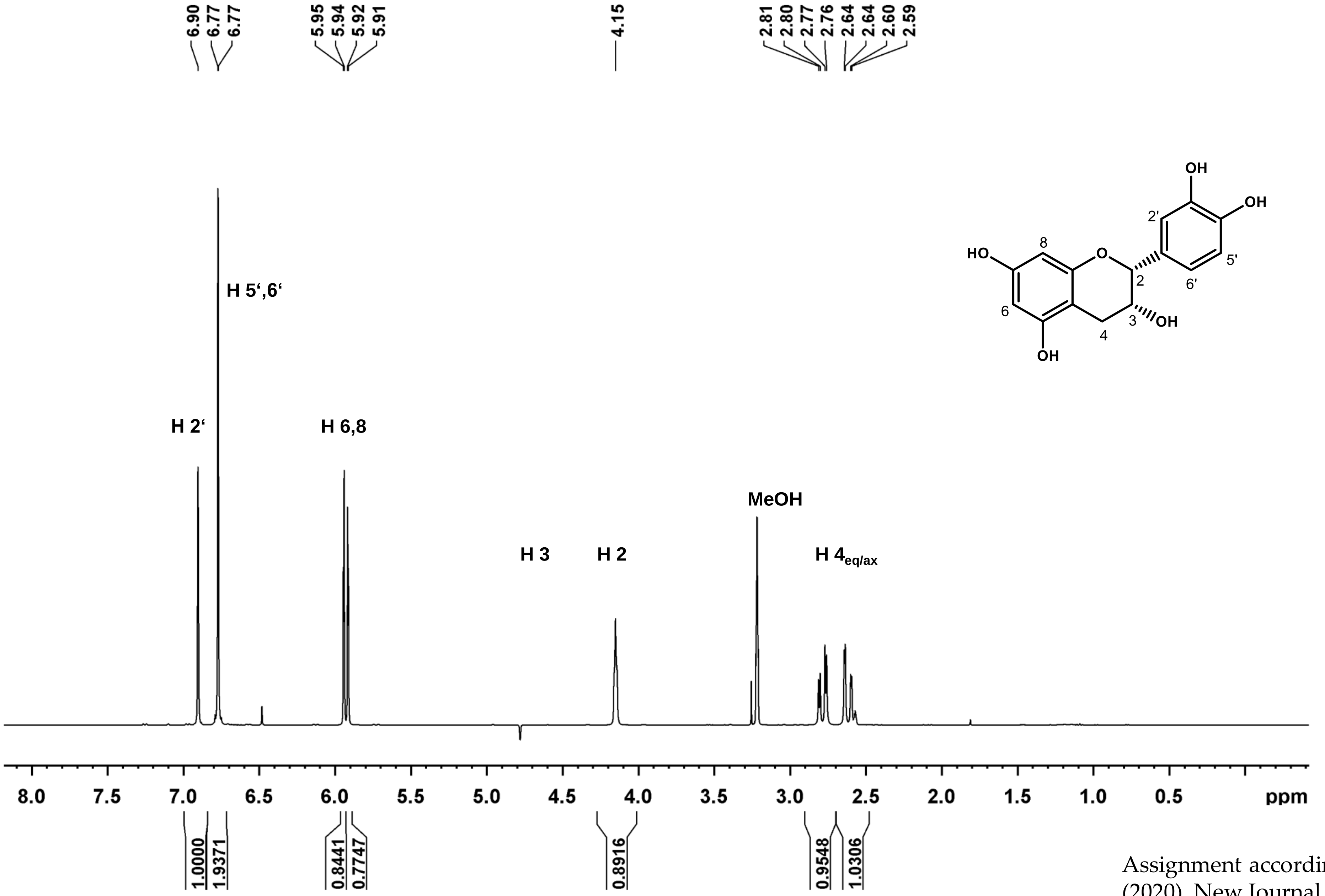

Assignment according to da Silva et al. (2020). New Journal of Chemistry, 44, 17391-17404.

procyanidin B1

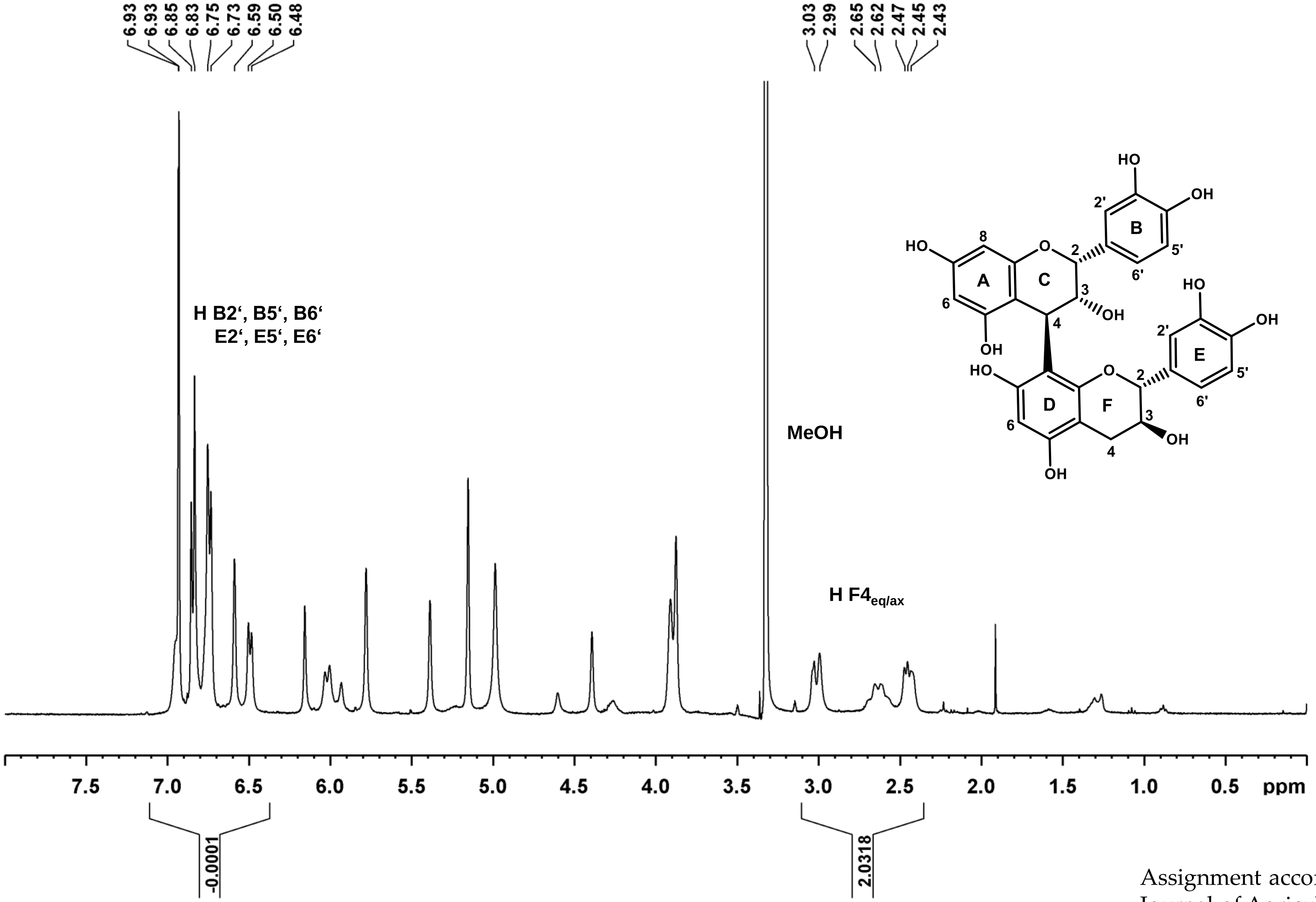

Assignment according to Shoji et al. (2003). Journal of Agricultural and Food Chemistry, 51, 3806-3813.

procyanidin B2

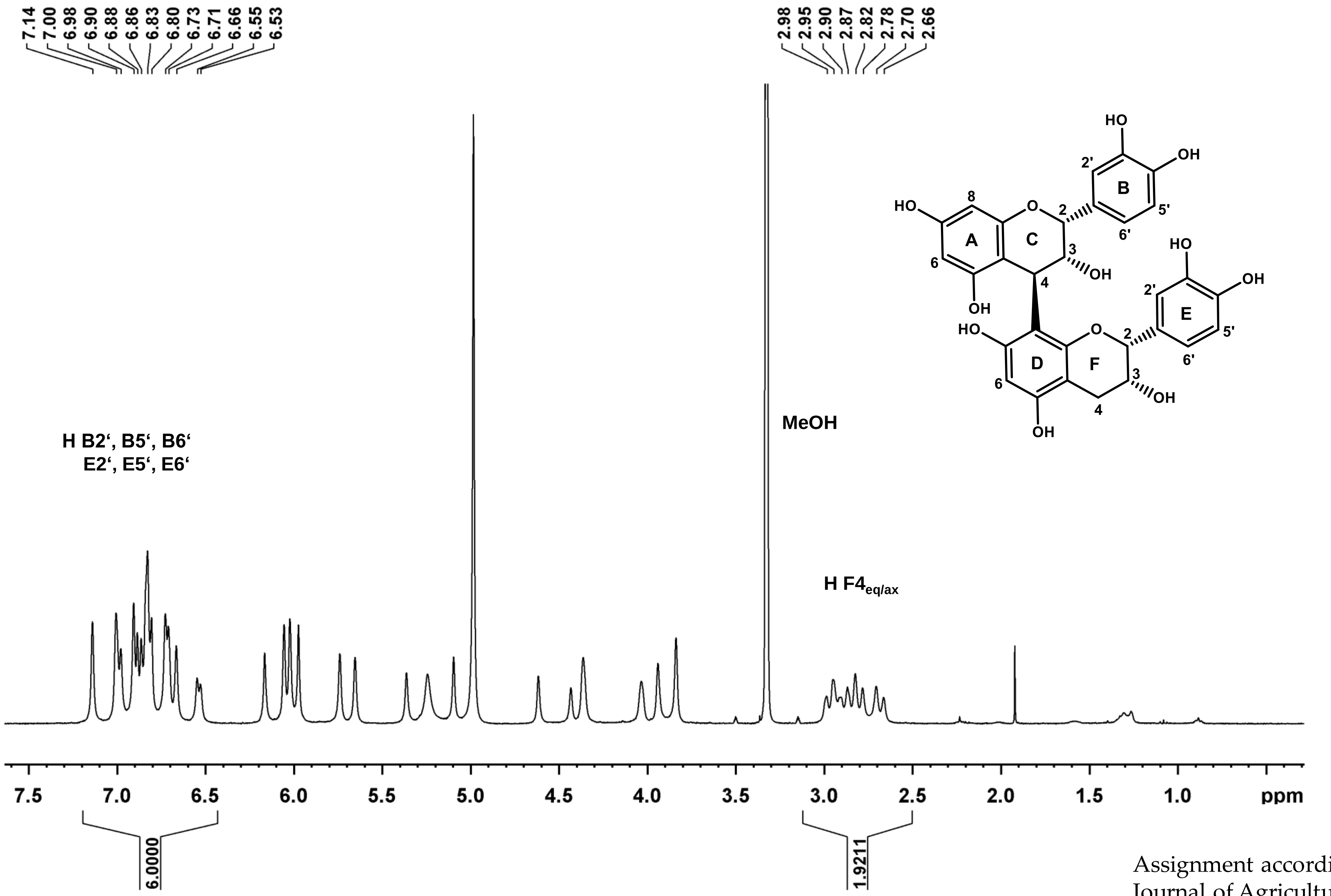

Assignment according to Shoji et al. (2003). Journal of Agricultural and Food Chemistry, 51, 3806-3813.

procyanidin C1

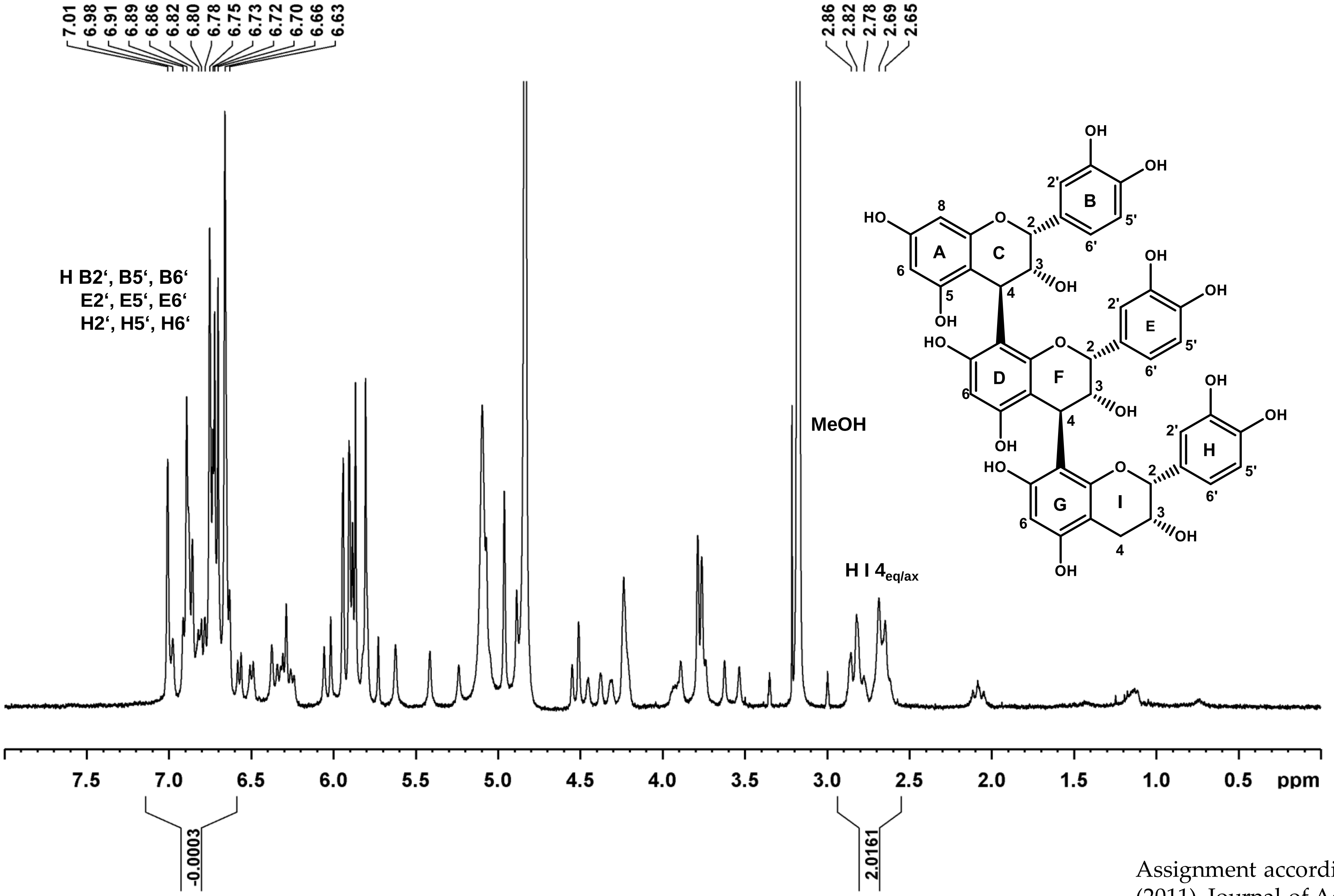

Assignment according to Esatbeyoglu et al. (2011). Journal of Agricultural and Food Chemistry, 59, 62–69.

quercetin-3-*O*-glucoside

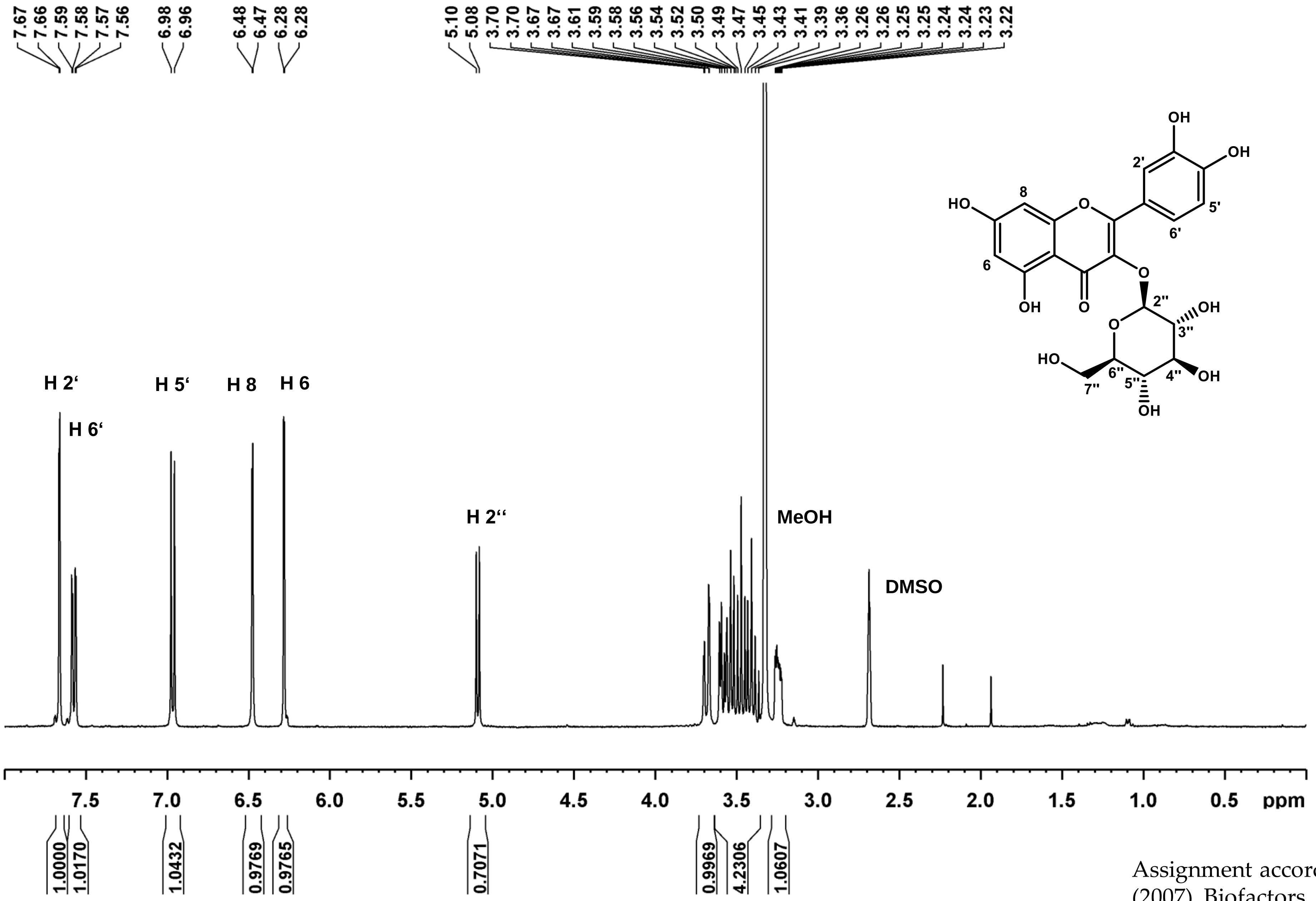

Assignment according to Panda and Kar (2007). Biofactors, 31, 201–210.

phlorizin

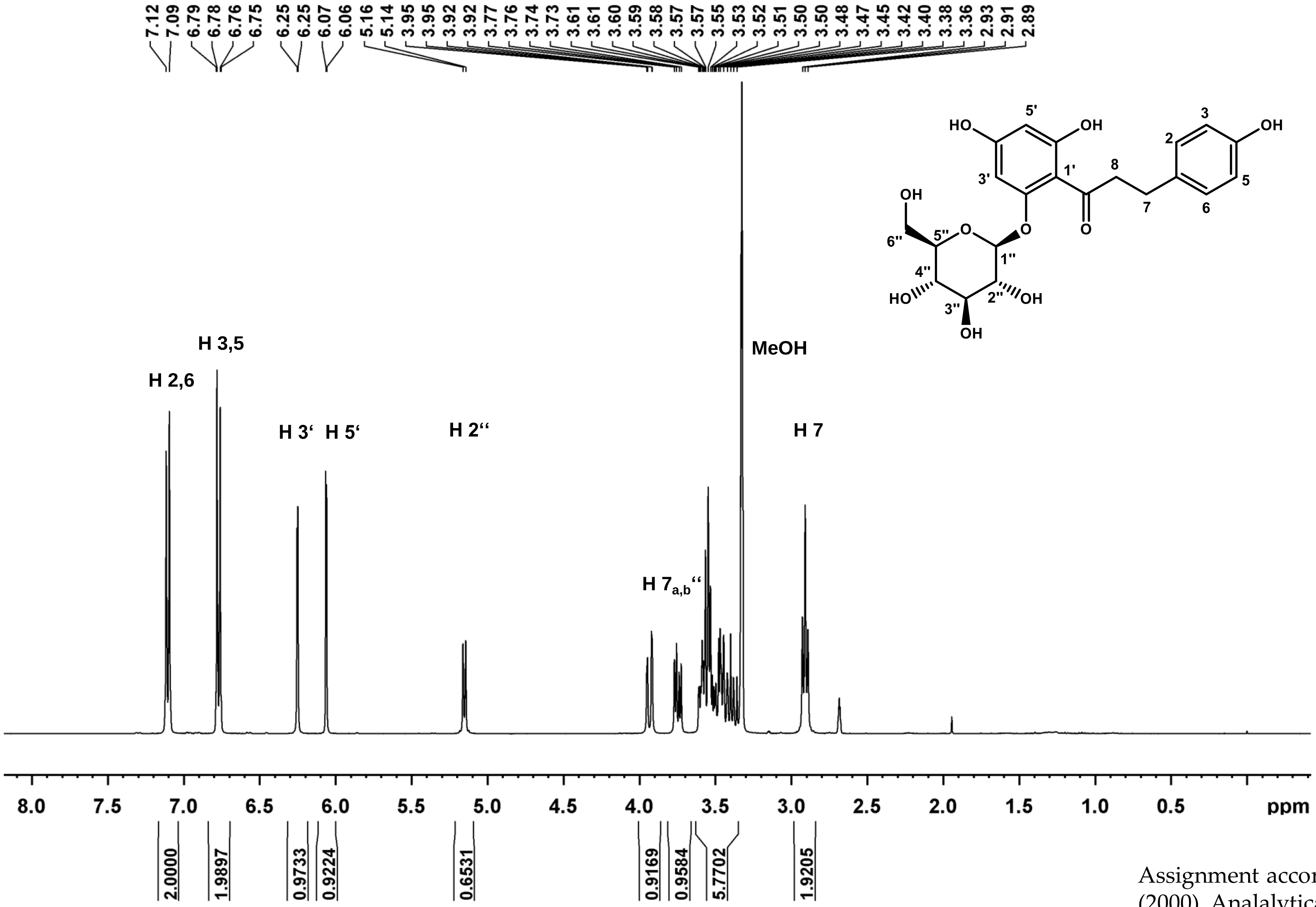

Assignment according to Lommen et al. (2000). Analalytical Chemistry, 2000, 72, 1793-1797.

isorhamnetin-3-*O*-rutinoside

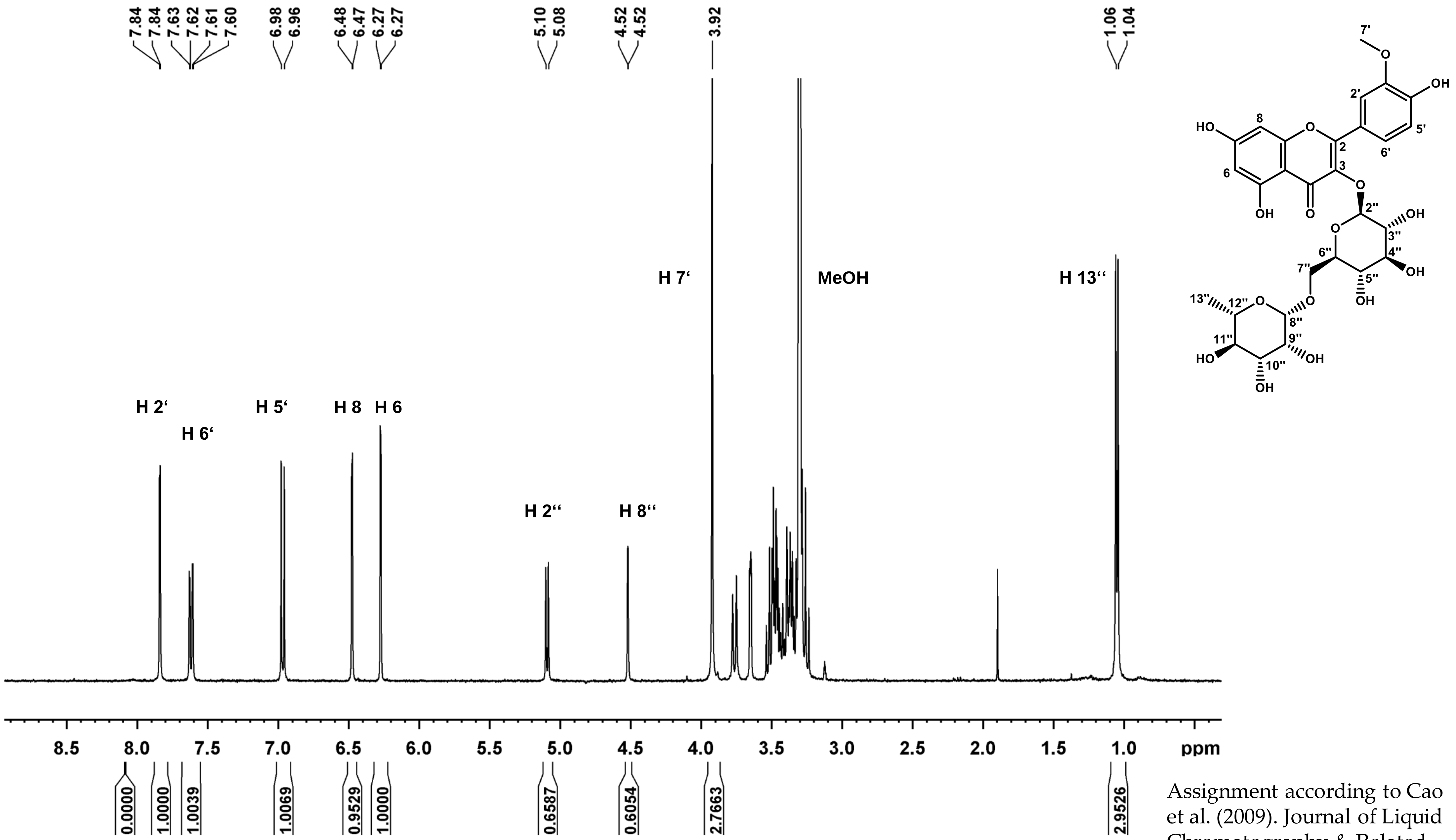

Assignment according to Cao et al. (2009). Journal of Liquid Chromatography & Related Technologies, 32, 273–280.

resveratrol

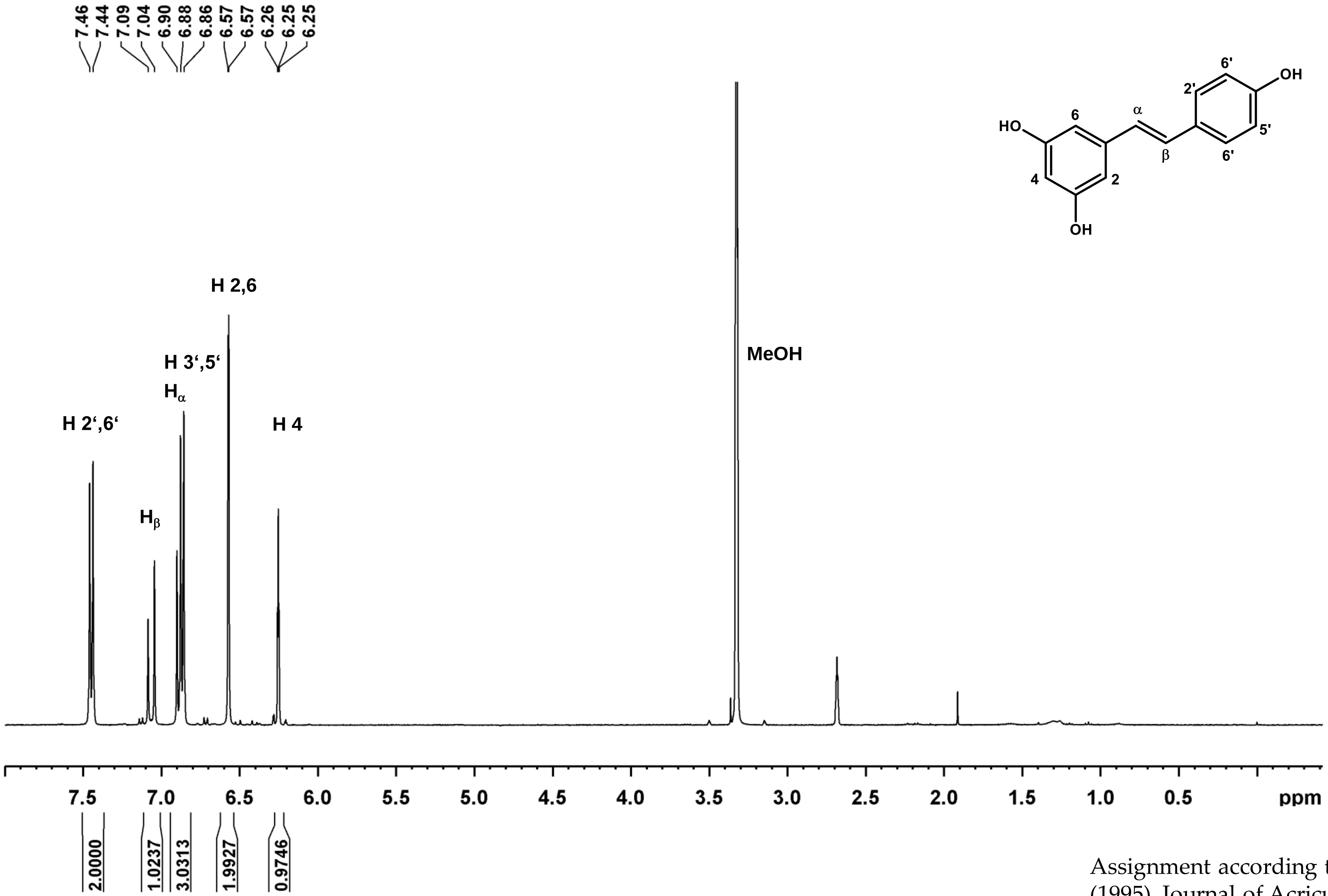

Assignment according to Mattivi et al. (1995). Journal of Acricultural and Food Chemistry, 43, 1820-1823.

EGCG

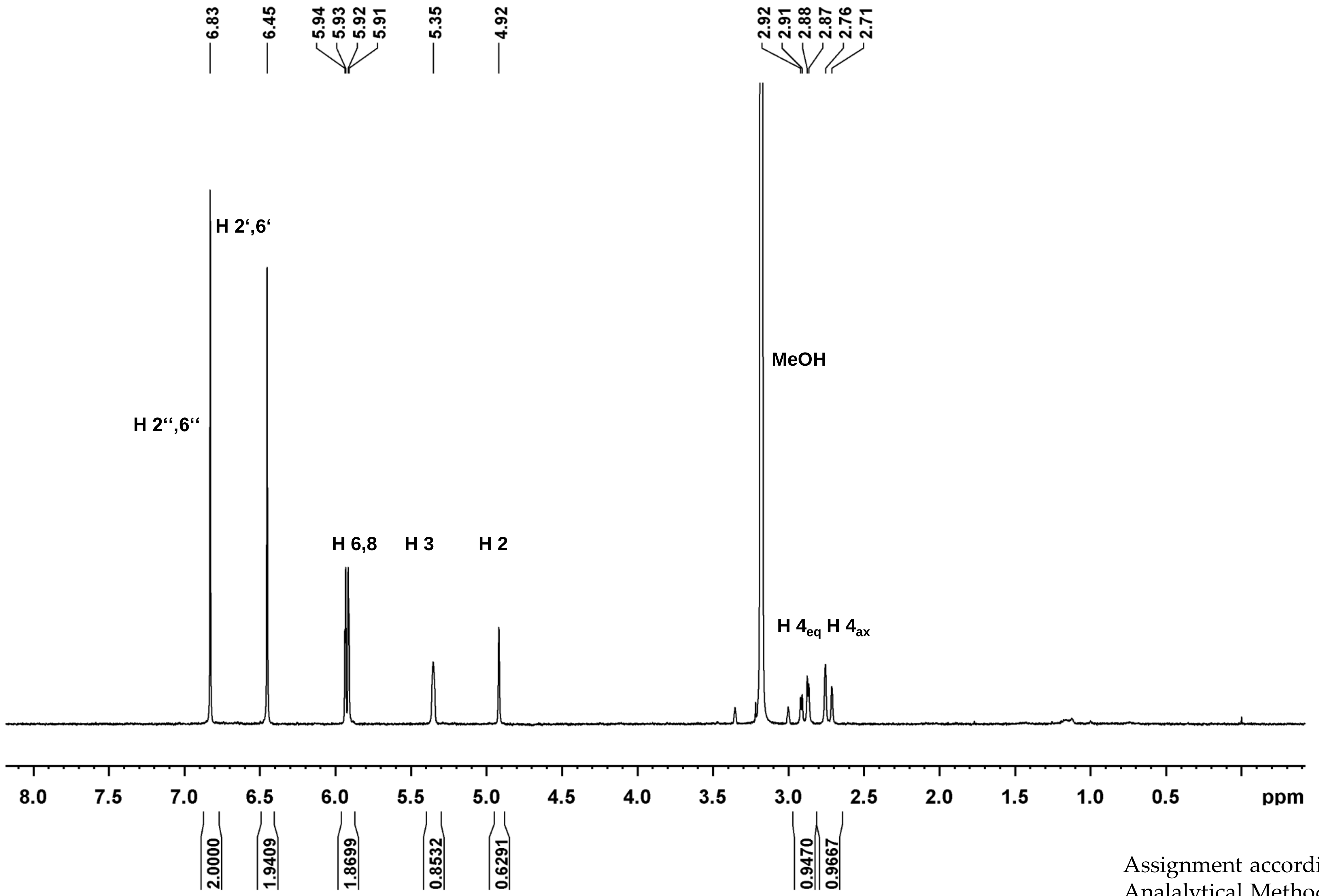

Assignment according to Yuan et al. (2014). Analalytical Methods, 6, 907-914.
